# An Analysis of Growth, Differentiation and Apoptosis Genes with Risk of Renal Cancer

**DOI:** 10.1371/journal.pone.0004895

**Published:** 2009-03-24

**Authors:** Linda M. Dong, Paul Brennan, Sara Karami, Rayjean J. Hung, Idan Menashe, Sonja I. Berndt, Meredith Yeager, Stephen Chanock, David Zaridze, Vsevolod Matveev, Vladimir Janout, Hellena Kollarova, Vladimir Bencko, Kendra Schwartz, Faith Davis, Marie Navratilova, Neonila Szeszenia-Dabrowska, Dana Mates, Joanne S. Colt, Ivana Holcatova, Paolo Boffetta, Nathaniel Rothman, Wong-Ho Chow, Philip S. Rosenberg, Lee E. Moore

**Affiliations:** 1 Division of Cancer Epidemiology and Genetics, National Cancer Institute, Bethesda, Maryland, United States of America; 2 International Agency for Research on Cancer, Lyon, France; 3 Samuel Lunenfeld Research Institute of Mount Sinai Hospital, Toronto, Canada; 4 Core Genotyping Facility, National Cancer Institute, Gaithersberg, Maryland, United States of America; 5 Institute of Carcinogenesis, Moscow, Russia; 6 Palacky University, Olomouc, Czech Republic; 7 Institute of Hygiene and Epidemiology, Prague, Czech Republic; 8 Department of Family Medicine and Public Health Sciences and Karmanos Cancer Institute, Wayne State University, Detroit, Michigan, United States of America; 9 Department of Biostatistics and Epidemiology, University of Illinois at Chicago, Chicago, Illinois, United States of America; 10 Masaryk Memorial Cancer Institute, Brno, Czech Republic; 11 Institute of Occupational Medicine, Lodz, Poland; 12 Institue of Public Health, Bucharest, Romania; Ohio State University Medical Center, United States of America

## Abstract

We conducted a case-control study of renal cancer (987 cases and 1298 controls) in Central and Eastern Europe and analyzed genomic DNA for 319 tagging single-nucleotide polymorphisms (SNPs) in 21 genes involved in cellular growth, differentiation and apoptosis using an Illumina Oligo Pool All (OPA). A haplotype-based method (sliding window analysis of consecutive SNPs) was used to identify chromosome regions of interest that remained significant at a false discovery rate of 10%. Subsequently, risk estimates were generated for regions with a high level of signal and individual SNPs by unconditional logistic regression adjusting for age, gender and study center. Three regions containing genes associated with renal cancer were identified: caspase 1/5/4/12(*CASP 1/5/4/12*), epidermal growth factor receptor (*EGFR*), and insulin-like growth factor binding protein-3 (*IGFBP3*). We observed that individuals with *CASP1/5/4/12* haplotype (spanning area upstream of *CASP1* through exon 2 of *CASP5*) GGGCTCAGT were at higher risk of renal cancer compared to individuals with the most common haplotype (OR:1.40, 95% CI:1.10–1.78, p-value = 0.007). Analysis of *EGFR* revealed three strong signals within intron 1, particularly a region centered around rs759158 with a global p = 0.006 (GGG: OR:1.26, 95% CI:1.04–1.53 and ATG: OR:1.55, 95% CI:1.14–2.11). A region in *IGFBP3* was also associated with increased risk (global p = 0.04). In addition, the number of statistically significant (p-value<0.05) SNP associations observed within these three genes was higher than would be expected by chance on a gene level. To our knowledge, this is the first study to evaluate these genes in relation to renal cancer and there is need to replicate and extend our findings. The specific regions associated with risk may have particular relevance for gene function and/or carcinogenesis. In conclusion, our evaluation has identified common genetic variants in *CASP1*, *CASP5*, *EGFR*, and *IGFBP3* that could be associated with renal cancer risk.

## Introduction

Renal cancer is among the most commonly diagnosed cancers in men and women in the United States [1] and Eastern Europe [2]. The incidence of renal cell carcinoma (RCC), the most common malignancy of renal cancer, has increased rapidly worldwide over the past few decades [3], [4] with some of the highest rates occurring in Central and Eastern Europe [2], [5]. Only a few well-established lifestyle risk factors have been identified: cigarette smoking, obesity, hypertension and diabetes [6]. An increased risk observed among those with a family history of renal cancer and the identification of inherited forms of kidney cancer provide justification for evaluating the genetic susceptibility of this disease, which has not been fully investigated [6].

The mechanism by which a normal cell progresses to carcinoma customarily involves the disruption of critical molecular pathways in cellular growth, differentiation, and development [7]. Among the steps required for tumor cell growth and survival are the amplification of signals from growth factors and the interruption of signals promoting cell death or apoptosis [8], [9]. Alterations in genes involved in such pathways are thus likely to contribute to cancer risk. Based on this logic, we identified genes involved in cell growth and differentiation (*AKR1C3*, *EGF*, *EGFR*, *IGFBP3*, *IGFBP5*, *PPARG*, *TGFA*, *VCAM1*, and *VEGF*) and apoptosis (*CASP1*, *CASP2*, *CASP3*, *CASP4*, *CASP5*, *CASP6*, *CASP7*, *CASP8*, *CASP9*, *CASP10*, *CASP12*, and *CASP14*; Table 1). Several of these genes have been associated with risk of cancer at other sites [10], [11]; however, the role of these genes in the development of renal cancer remains unknown.

**Table 1:** Description of selected genes and tagging SNPs.

Name of Gene	Function	Chromosome Location	Number of tagSNPs^1^/tagSNPs p<0.05^2^	Adjusted min P test
Growth and Differentiation Genes				
*AKR1C3* - Aldo-keto Reductase Family 1, Member C3	Catalyzes the reduction of prostaglandin (PGD). Role in cell differentiation by diverting conversion of PGD from PGJ2 to PGF2.	10p15-p14	17/1	0.57
*EGFR* -Epidermal Growth Factor Receptor	A transmembrane growth factor receptor. EGFR binding leads to the activation of major signal transduction pathways involved in regulating cell proliferation, differentiation, migration and survival.	7p12	65/12*	0.48
*EGF* - Epidermal Growth Factor	EGF is a mitogenic factor and an important ligand for EGFR. Subsequently has a major effect on cell differentiation.	4q25	16/0	0.90
*IGFBP3* - Insulin-like Growth Factor Binding Protein 3	Main carrier of circulating IGFs. Independently, has a key role in regulating cell proliferation and apoptosis.	7p13-p12	14/3*	0.23
*IGFBP5* - Insulin-like Growth Factor Binding Protein 5	Another carrier for IGF. Role in regulating cell survival, differentiation, and apoptosis.	2q33-q36	17/1	0.34
*PPARG*- Peroxisome Proliferator-activated Receptor Gamma	A nuclear hormone receptor. When dimerizes with retinoid X receptor, regulates transcription of numerous genes. Also a regulator of adipocyte differentiation	3p25	26/1	0.66
*TGFA* -Transforming Growth Factor, alpha	Growth factor that competes with EGF in binding to EGFR	2p13	39/1	0.76
*VCAM1* - Vascular Cell Adhesion Molecule 1	Member of the immunoglobulin family and encodes a cell surface glycoprotein expressed by cytokine-activated endothelial cells	1p32-p31	14/1	0.41
*VEGF* - Vascular Endothelial Growth Factor A	Important growth factor in angiogenesis and tumor growth	6p12	21/1	0.78
Apoptosis Genes				
*CASP1/5/4/12*	A member of the cysteine-aspartic acid protease (caspase) family which plays an integral role in a complex cascade of events that regulate cell apoptosis	11q22.2-22.3	24/5*	0.26
*CASP2*	Member of the caspase family	7q34-q35	6/0	0.90
*CASP3*	Member of the caspase family	4q34	10/0	0.83
*CASP6*	Member of the caspase family	4q25	7/0	0.77
*CASP7*	Member of the caspase family	10q25	16/0	0.85
*CASP8/10*	Member of the caspase family	2q33-q34	12/0	0.85
*CASP9*	Member of the caspase family	1p36.3-p36.1	7/0	0.96
*CASP14*	Member of the caspase family	19p13	8/1	0.25

1 rs numbers for SNPs can be found in supplementary table 1.

2 TagSNPs with p-value for trend <0.05.

* indicates genes where number of tagSNPs with p-value for trend <0.05 is more than one would expect to see by chance.

Given the importance of these pathways in carcinogenesis and the lack of studies evaluating genetic susceptibility and renal cancer, we evaluated whether polymorphisms in these 21 genes could alter the risk for developing renal cancer in a large multi-center case-control study based in Central and Eastern Europe. We hypothesized that common variation in genes involved in cellular growth, differentiation and apoptosis may increase genetic susceptibility to renal cancer.

## Methods

### Study Population

The Central and Eastern European Renal Cancer (CEERC) Study is a hospital-based case-control study of renal cancer (1,097 cases and 1,555 controls) that was conducted in seven centers in Eastern and Central Europe (Moscow, Russia; Bucharest, Romania; Lodz, Poland; and Prague, Olomouc, Ceske Budejovice and Brno, Czech Republic). Details of the study have been described previously [12]. Newly diagnosed and histologically confirmed cases of renal cancer (ICD-0-2 code C64) between the ages of 20 and 79 years were recruited from August 1999 through January 2003. Trained medical staff reviewed medical records and extracted information on date and method of diagnosis, histological classification, tumor location, stage and grade. Pathology data was available for 917 cases. RCC was defined as the following subtypes: clear cell, clear cell with papillary features, clear cell with sarcomatoid, papillary type I, papillary non-type I, papillary type II, chromophobe and hybrid subtype (n = 848). Clear cell renal cancer was defined as the first three clear cell subtypes (n = 760). Eligible controls were chosen from among patients admitted to the same hospital as cases for conditions unrelated to smoking or genitourinary disorders (except for benign prostatic hyperplasia) and were frequency-matched to cases on age (within 3 years), sex, and study center. Among controls, the disease conditions associated with hospitalization were the following: obstetric or perinatal (0.1%), infectious (1%), psychiatric (1%), endocrine (2%), hematologic (3%), dermatologic (3%), injury or poisoning (3%), genitourinary (benign prostatic hyperplasia (4%), pulmonary (4%), orthopedic or rheumatologic (9%), cardiovascular (10%), neurologic (11%), ophthalmologic or otologic (14%), gastrointestinal (19%), and other (16%). No single disease made up more than 20% of the control group. A portion of the controls were also recruited for a parallel study of lung cancer. All recruited cases and controls were Caucasian. Response rates at each center ranged from 90.0 to 98.6% for cases and from 90.3 to 96.1% for controls.

Interviews were conducted by trained personnel to collect standardized lifestyle and food frequency questionnaires. Data was collected on demographic characteristics, education, tobacco smoke exposures, alcohol consumption, dietary practices, anthropometry, medical history, family history, and occupational history.

Blood samples were collected and stored at −80°C and shipped to the National Cancer Institute (NCI). Genomic DNA was extracted from whole blood buffy coat by the standard phenol chloroform method at the NCI laboratory. All subjects in this study provided written informed consent. This study was approved by the institutional review boards (IRB) at the NCI, International Agency for Research on Cancer (IARC), and each participating center.

### Genotyping

We analyzed 319 tagging single-nucleotide polymorphisms (SNPs) in 21 genes involved in cellular growth, differentiation (*AKR1C3*, *EGFR*, *EGF*, *IGFBP3*, *IGFBP5*, *PPARG*, *TGFA*, *VCAM1*, *VEGF*) and apoptosis (*CASP1*, *CASP5*, *CASP4*, *CASP12*, *CASP2*, *CASP3*, *CASP6*, *CASP7*, *CASP8*, *CASP10*, *CASP9*, *CASP14*) that we hypothesized could increase RCC risk (Table 1). Since several of the caspase genes were located relatively close to each other, tagSNPs were chosen to comprehensively assess the variation in the region rather than just the gene (*CASP1/5/4/12* and *CASP8/10*). TagSNPs were selected from among common variants (minor allele frequencies ≥5%) found in Caucasians using a tagSNP method [13] with an r^2^>0.80 to provide high genomic coverage. In addition, important nonsynonymous SNPs or those with potential functional significance were included. All SNPs are reported in the NCI SNP500Cancer database (http://snp500cancer.nci.nih.gov)[14]. Genotyping was conducted at NCI's Core Genotyping Facility where staff was blinded to case/control status and duplicate quality control samples (5% samples) interspersed among plates. All genotyping was performed using an Illumina GoldenGate ® Oligo Pool All (OPA) assay, which was designed using publicly available sequencing information. The genotype frequencies among controls showed no deviation from the expected Hardy-Weinberg equilibrium proportions (p>0.05). All SNPs had a genotyping completion rate >98% except for rs3770472 (96%). The quality control concordance rates were >97% for all SNPs except for rs10885493 (92%), rs12416109 (92%), rs10752001 (94%), and rs13392762 (94%).

### Replication Study

The US Kidney Cancer Study is a population-based case-control study conducted in Detroit and Chicago. Cases were residents of the study areas, aged 20 to 79 years who were newly diagnosed with histologically confirmed renal cell carcinoma (ICD-O2 C64.9) from February 2002 through January 2007. Controls were frequency-matched to cases by study center, race, age, and sex. Controls aged 65 years and older were identified from Medicare files, and those under age 65 years were identified from Division of Motor Vehicle records. African American cases and controls were over-sampled. Written informed consent was obtained from all participants, and IRB approvals were obtained from all participating study centers.

Participants were interviewed by trained interviewers to elicit information on demographic factors, use of tobacco and alcohol, diet, occupational history, height and weight history, family history of cancer, reproductive history among women, medical history, and medication history including the use of diet pills and antihypertensives. A total of 1568 Caucasians (856 cases and 712 controls) and 884 African-Americans (523 cases and 361 controls) were interviewed. Of these subjects, 1109 cases and 1106 controls provided DNA that was extracted using standard procedures. Genotyping data was available for 966 cases and 977 controls with sufficient quality and quantity of DNA. Subjects were predominantly recruited from the Detroit area (84%), and were similar in age (76%>50 years) and sex (57% male) to those in the CEERC study.

### Statistical Analyses

Of the 987 cases and 1298 controls that had valid study data and provided genomic DNA, analyses were based on the 777 cases and 1035 controls that had adequate quality DNA and were successfully genotyped on the OPA platform in the CEERC study. Associations were evaluated through several methods. Global p-values were evaluated using the minimum-p value permutation test [15]. A haplotype-based method called HaploWalk, conducted in Matlab, was used to identify chromosome regions of interest by examining regional associations rather than effects from an individual SNP. For a gene with K SNPs, the HaploWalk procedure considered a 3 SNP sliding window for each SNP from SNP 2 through SNP K-1. To account for multiple testing across the *K* SNPs, the *K-2* p-values (one for each window) were adjusted for multiple comparisons using the False Discovery Rate (FDR)-controlling procedure of Benjamini and Hochberg [16]. Windows that remained significant at a FDR level of 10% were considered to be a candidate region of interest. If adjacent windows were significant, they were amalgamated into a single candidate region of interest. Haplotypes in the candidate block were then reconstructed and effects evaluated using Haplostats (Version 1.3.1) in R (version 2.4.1). The most common haplotype was used as the reference group and haplotypes with frequencies less than 1% were combined into one category for testing. Subsequently, unadjusted and adjusted (age, sex, and study center) odds ratios (OR) and 95% confidence intervals (95% CI) using the log-additive model were generated for regions with a high level of signal.

The association between individual SNPs and risk of renal cancer were estimated by unconditional logistic regression, adjusted for age, sex, and study center. Genotypes were evaluated by coding the homozygous common allele as the referent group and separately comparing the heterozygous and homozygous rare allele genotypes to the referent group. Linear tests for trends were conducted by including a variable coded 0, 1, and 2 corresponding to the number of rare alleles. Associations for SNPs were considered robust if they were significant (based on the p-value of the test for trend) with a FDR level of 20% or less. A more liberal FDR level was chosen at this stage of analysis in order to guide us toward SNPs that may be of interest within previously identified regions of interest. FDR adjustment was based on the number of SNPs within each gene region. Additional adjustment for potential confounders (body mass index [BMI], self-reported hypertension, and smoking) did not result in meaningful changes of the risk estimates and were not included in the analyses. In addition, we investigated multiplicative interaction between individual SNPs and age, sex, and BMI, using the likelihood ratio test to compare the fit of models with and without interaction terms. Heterogeneity of genotype frequencies among countries was evaluated by using the likelihood ratio test to compare the fit of models with and without interaction terms, but we did not find any evidence of heterogeneity. Analyses were conducted using SAS version 9.1 (SAS Institute, Cary, NC).

## Results

A large proportion of the study population was from the Czech Republic, with a slightly higher proportion among cases (Table 2). Controls were more likely to be male, but were similar to cases in age distribution. Cases were more likely than controls to have higher BMI, have a family history of cancer, and report hypertension.

**Table 2:** Distribution of demographic variables among subjects in the Central and Eastern European Renal Cancer study.

	Cases		Controls		p-value
	N	%	N	%	
All subjects	987		1298		
Center					
Bucharest, Romania	91	9.2	132	10.2	
Lodz, Poland	81	8.2	197	15.2	
Moscow, Russia	288	29.2	368	28.4	
Czech Republic^1^	527	53.4	601	46.3	<.0001
Sex					
Male	589	59.7	838	64.6	
Female	398	40.3	460	35.4	0.02
Age at Interview (y)					
<50	163	16.5	228	17.6	
≥50	824	83.5	1070	82.4	0.51
Smoking Status					
Never	454	46.1	528	40.7	
Former	225	22.9	316	24.4	
Current	305	31.0	452	34.9	0.03
Body Mass Index (kg/m^2^)					
<25	288	29.2	457	35.4	
25–30	429	43.5	556	43.1	
30+	270	27.4	278	21.5	0.001
Family history of cancer^2^					
No	654	66.3	932	71.8	
Yes	333	33.7	366	28.2	0.004
Self-Reported Hypertension					
No	539	54.7	800	61.7	
Yes	447	45.3	497	38.3	0.001

1 Four centers: Brno, Olomuc, Prague, and Ceske.

2 First degree relative with any cancer.

Results from global gene-based tests of association are included in Table 1. Among results from the minimum p-value test, *CASP1/5/4/12*, *CASP14*, and *IGFBP3* were the most promising gene regions, but were not significant after adjustment for multiplicity (total number of SNPs) over the entire gene (Table 1). However, *CASP1/5/4/12*, *EGFR*, and *IGFBP3* had a larger number of significant SNPs (p-value for trend <0.05) than one would expect to see by chance. In addition, with a haplotype-based sliding window method, we identified the same genes with regions that were associated with renal cancer risk at a FDR level <10%: *CASP1/5/4/12*, *EGFR*, *IGFBP3*, and *VCAM1* (Supplementary Figures S1, S2, S3).

An interesting region was detected that spans over the area upstream of *CASP1* through exon 2 of *CASP5* (Supplementary Figure S1). At this region, individuals with a specific variant haplotype GGGCTCAGT (OR: 1.40, 95% CI: 1.10–1.78) had a 1.4 fold higher risk of renal cancer compared to those with the most common haplotype (Table 3). Concordant with the haplotype analysis, several individual variants within this haplotype also had nominal statistically significant associations with renal cancer risk (Table 4). After applying FDR adjustment, four *CASP1* and *CASP5* SNPs (rs1785883, rs568910, rs492859 and rs507879) were considered significant at a FDR level <20%. The strongest association among individual SNPs was rs507879 (Thr90Ala), located in exon 2 of *CASP5*. The ORs (95% CI) for heterozygote and homozygote rare genotypes compared to the homozygote common genotypes were 1.29 (1.03–1.60) and 1.39 (1.07–1.82; p-value for trend = 0.01), respectively. The OR and p-value of the specific variant haplotype were stronger than the associations (p-value for trend) observed for any of the individual SNPs in this region, suggesting that the causal variant within this haplotype may not have been genotyped.

**Table 3:** Haplotype associations and Renal Cancer Risk.

Gene/Haplotypes	Cases (%)	Controls (%)	OR^1^	95% CI	Unadjusted p-value	Adjusted p-value^1^
***CASP1/5/4/12*** ** haplotype**						
Region 1 (chr11: 104429456-104390522)						
G-G-G-T-T-A-C-G-T	41.8	45.0	1.00			
G-G-G-T-C-A-C-A-C	28.1	26.7	1.14	0.97–1.34		0.12
G-G-G-C-T-C-A-G-C	10.6	8.1	**1.40**	**1.10–1.78**		**0.007**
A-G-G-T-T-A-C-G-T	5.5	7.4	0.80	0.60–1.07		0.13
Global p-value					0.14	0.16
***EGFR*** ** haplotype**						
Region 1 (chr7 55122130-55123913)						
C-G-T	36.8	36.0	1.00			
C-A-A	21.8	24.7	0.87	0.73–1.03		0.09
T-G-T	20.1	21.2	0.93	0.78–1.12		0.20
C-G-A	21.4	18.1	1.13	0.94–1.36		0.40
Global p-value					**0.04**	0.06
Region 2 (chr 7 55129830-55138684)						
C-G-G	29.5	29.9	1.00			
T-A-G	25.0	25.9	0.99	0.82–1.18		0.88
T-G-G	16.7	17.3	1.00	0.79–1.26		0.98
C-A-G	11.3	12.1	0.94	0.72–1.24		0.67
T-G-A	11.1	8.5	**1.32**	**1.02–1.70**		**0.03**
C-G-A	6.4	6.4	1.07	0.76–1.52		0.70
Global p-value					0.31	0.30
Region 3 (chr 7 55143370-55147338)						
A-T-G	34.4	38.4	1.00			
A-G-G	22.6	22.7	1.11	0.91–1.34		0.30
G-G-G	20.8	18.7	**1.26**	**1.04–1.53**		**0.02**
A-T-A	12.3	11.8	1.14	0.90–1.43		0.28
G-T-G	9.7	7.0	**1.55**	**1.14–2.11**		**0.005**
Global p-value					**0.007**	**0.006**
***IGFBP3*** ** haplotype**						
Region 1 (chr 7 45940583-45921554)						
T-A-A-T-T-C-A-G	38.5	38.1	1.00			
T-A-A-G-C-C-A-A	19.8	21.5	0.94	0.78–1.13		0.52
T-A-A-T-C-C-A-G	15.3	17.9	0.87	0.71–1.05		0.15
T-A-G-T-C-G-A-A	19.6	16.9	1.17	0.98–1.41		0.08
T-G-A-T-T-C-A-G	3.6	3.3	1.07	0.73–1.58		0.73
A-A-G-T-C-G-A-A	1.3	1.0	1.13	0.60–2.11		0.70
Global p-value					0.19	0.21
Region 2 (chr 7 45918779-45916095)						
T-G-C	48.6	51.3	1.00			
A-G-C	18.6	15.2	**1.27**	**1.06–1.54**		**0.01**
T-G-T	13.8	15.4	0.93	0.75–1.16		0.54
T-A-C	14.3	14.5	1.02	0.82–1.27		0.83
T-A-T	4.7	3.1	**1.62**	**1.05–2.51**		**0.03**
Global p-value					**0.03**	**0.04**
***VCAM1*** ** haplotype**						
Region 1 (chr 1: 100961998-100966793)						
C-A-C	45.3	45.9	1.00			
C-C-C	18.8	17.4	1.10	0.89–1.35		0.40
C-A-T	20.3	16.5	**1.25**	**1.01–1.54**		**0.04**
T-A-C	8.3	10.6	0.81	0.63–1.04		0.09
C-C-T	6.1	8.6	0.75	0.55–1.02		0.07
Global p-value					**0.02**	**0.03**

SNPs included within haplotype regions:

*CASP1/5/4/12*: region 1: rs1785883, rs508760, rs7934239, rs501626, rs11821722, rs568910, rs492859, rs3181318, rs507879.

*EGFR*: region 1: rs759169, rs11238349, rs12535226; region 2: rs11977660, rs6593205, rs6954351; region 3: rs7796139, rs759158, rs7796872.

*IGFBP3*: region 1: rs10235181, rs13232606, rs2453836, rs903889, rs924140, rs2471551, rs9282734, rs3110697; region 2: rs6670, rs13223993, rs2270628.

*VCAM1* chr1: region 1: rs3917009, rs3917010, rs3176867.

1 Adjusted for age, sex, and center.

**Table 4:** Association between Selected Polymorphisms and Renal Cancer Risk.

SNP/Genotypes	Cases	Controls	OR^1^	95% CI		p-trend
***CASP 1/5/4/12***						
*CASP1*						
rs1785883 (*12058T>C)						
GG	686	880	1.00			
AG	85	145	**0.75**	**0.56**	**1.00**	
AA	2	8	0.34	0.07	1.60	**0.02**
AG+AA			**0.75**	**0.57**	**1.00**	
rs508760 (*12353A>C)						
GG	658	893	1.00			
GT	112	135	1.12	0.85	1.47	
TT	4	6	1.05	0.29	3.81	0.45
GT+TT			1.13	0.87	1.48	
rs7934239 (−12676T>C)						
GG	700	940	1.00			
AG	74	90	1.11	0.80	1.54	
AA	2	2	1.27	0.18	9.10	0.50
AG+AA			1.09	0.79	1.50	
rs501626 (−12291A>G)						
TT	580	820	1.00			
CT	183	199	**1.30**	**1.04**	**1.64**	
CC	14	16	1.19	0.57	2.48	**0.03**
CT+CC			**1.29**	**1.04**	**1.62**	
rs11821722 (−11804G>A )						
TT	396	548	1.00			
CT	310	405	1.09	0.89	1.33	
CC	70	81	1.21	0.85	1.71	0.23
CT+CC			1.11	0.92	1.34	
rs568910 (IVS2+365T>G)						
AA	518	744	1.00			
AC	225	254	**1.28**	**1.03**	**1.59**	
CC	31	35	1.24	0.75	2.05	0.03
AC+CC			**1.28**	**1.05**	**1.57**	
*CASP5*						
rs492859 (−5645T>G)						
CC	514	741	1.00			
AC	227	251	**1.31**	**1.06**	**1.62**	
AA	30	35	1.21	0.73	2.00	**0.02**
AC+AA			**1.29**	**1.05**	**1.58**	
rs3181318 (−373 C>T)						
GG	337	472	1.00			
AG	349	457	1.09	0.90	1.33	
AA	89	106	1.20	0.88	1.65	0.21
AG+AA			1.12	0.93	1.35	
rs507879 (Ex2-118A>G, T90A)						
TT	212	337	1.00			
CT	381	481	**1.29**	**1.03**	**1.60**	
CC	179	209	**1.39**	**1.07**	**1.82**	**0.01**
CT+CC			**1.31**	**1.07**	**1.61**	
***EGFR***						
rs11977660 (IVS1-47643T>C)						
TT	220	285	1.00			
CT	381	499	0.98	0.79	1.23	
CC	176	251	0.88	0.68	1.15	0.37
CT+CC			0.95	0.77	1.17	
rs6593205 (IVS1-41287A>G)						
GG	310	414	1.00			
AG	368	456	1.09	0.89	1.34	
AA	98	164	0.80	0.60	1.07	0.37
AG+AA			1.02	0.84	1.23	
rs6954351 (IVS1-38789G>A)						
GG	522	752	1.00			
AG	236	257	**1.34**	**1.08**	**1.65**	
AA	18	26	1.04	0.56	1.93	**0.03**
AG+AA			**1.31**	**1.07**	**1.61**	
rs7796139 (IVS1-34103A>G)						
AA	370	543	1.00			
AG	337	423	1.17	0.96	1.42	
GG	70	68	**1.52**	**1.06**	**2.18**	**0.02**
AG+GG			**1.21**	**1.00**	**1.46**	
rs759158 (IVS1-30770G>T)						
TT	253	351	1.00			
GT	372	502	1.03	0.83	1.27	
GG	151	181	1.16	0.88	1.52	0.33
GT+GG			1.06	0.87	1.30	
rs7796872 (IVS1-30135G>A)						
GG	593	780	1.00			
AG	170	236	0.94	0.75	1.18	
AA	11	18	0.76	0.36	1.63	0.43
AG+AA			0.94	0.75	1.17	
***IGFBP3***						
rs6670 (Ex5-411A>T)						
TT	514	740	1.00			
AT	227	261	**1.24**	**1.00**	**1.53**	
AA	30	31	1.40	0.84	2.36	**0.03**
AT+AA			**1.27**	**1.04**	**1.56**	
rs13223993 (Ex5+615C>T )						
GG	506	702	1.00			
AG	247	294	1.15	0.94	1.42	
AA	24	37	0.87	0.51	1.49	0.46
AG+AA			1.11	0.91	1.36	
rs2270628 (4848 bp 3′ of STPG>A)						
CC	514	676	1.00			
CT	230	319	0.94	0.77	1.16	
TT	27	34	1.04	0.62	1.76	0.73
CT+TT			0.95	0.78	1.16	

1 Adjusted for age, sex, and center.

We had the opportunity to conduct a quick replication of our most statistically significant finding, *CASP5* SNP rs507879 in the US Kidney Cancer Study population (Table 5). Although results from the US Kidney Cancer Study were not statistically significant, the point estimates were in the same direction as those from the CEERC study. A pooled estimate of 1.22 (95% CI: 1.04–1.42) was observed for those with at least one copy of the rare allele of rs507879 among Caucasian participants. A pooled estimate including both Caucasians and African-Americans from both studies was not noticeably different from the estimate restricted to Caucasians (OR: 1.22, 95% CI: 1.05–1.41; Table 5).

**Table 5:** Results from Replication of SNP rs507879 and Renal Cancer Risk.

SNP/Genotypes	CEERC^1^				US Kidney (Whites only)^1^				Combined (Whites only)^1^				Combined (All)^2^^,^ 3			
	Cases	Controls	OR	95% CI	Cases	Controls	OR	95% CI	Cases	Controls	OR^1^	95% CI	Cases	Controls	OR^1^	95% CI
*CASP 1/5/4/12*																
rs507879 (T90A)																
TT	212	337	1.00		208	190	1.00		420	527	1.00		458	590	1.00	
CT	381	481	**1.29**	**1.03–1.60**	334	276	1.10	0.85–1.42	715	757	**1.20**	**1.01–1.41**	840	944	**1.19**	**1.02–1.39**
CC	179	209	**1.39**	**1.07–1.82**	154	127	1.10	0.80–1.49	333	336	**1.25**	**1.02–1.53**	440	472	**1.28**	**1.07–1.54**
CT+CC			**1.31**	**1.07–1.61**			1.10	0.87–1.39			**1.22**	**1.04–1.42**			**1.22**	**1.05–1.41**
p-trend				**0.01**				0.53				**0.02**				**0.007**

1 Adjusted for age, sex and study center.

2 Association among African-Americans in US Kidney Cancer Study (n = 270 cases and 384 controls, CT: OR: 1.23 (0.77–1.97); CC: OR: 1.37 (0.85–2.23); p-trend = 0.21.

3 Adjusted for age, sex, center and race.

A sliding window analysis over *EGFR* revealed three signals within intron 1 (Supplementary Figure S2). In particular, two haplotypes centered on rs759158 (region 3) were associated with a higher risk of renal cancer (GGG: OR: 1.26, 95% CI: 1.04–1.53 and ATG: OR: 1.55, 95% CI: 1.14–2.11; Table 3) when compared to the common haplotype. In the second *EGFR* region, variant haplotype TGA was associated with an increased risk of renal cancer compared to the common haplotype (OR: 1.32, 95% CI: 1.02–1.70). Associations between three of the SNPs within these *EGFR* haplotypes (rs11238349, rs6954351, and rs7796139) were nominally statistically significant, but with FDR levels <30% (Table 4). The two SNPs rs6954351 and rs7796139 appear to be responsible for the associations in their respective regions; however, these associations do not appear to be entirely independent effects as the SNPs are moderately correlated (r^2^ = 0.47). We further evaluated the strong signal in *EGFR* by integrating the second and third regions to form a haplotype spanning seven SNPs in intron 1 (Supplementary Table S2). Among common haplotypes, the effect estimates for haplotypes containing GGG or GTG appear to be consistently above 1.0. It is interesting to note that among common haplotypes in the integrated region, the variant haplotype TGA from the second region is present only with either variant haplotype GGG or GTG, the statistically significant haplotypes from the third region. This suggests that the two sets of haplotypes may be reflecting the same signal. A strong haplotype effect was observed for the variant haplotype TGA-A-GGG, with an OR of 1.84 (95% CI: 1.25–2.71) and a p-value of 0.002. This effect was stronger than those observed for the individual regions and reinforces the idea that these two regions are related. A second variant haplotype in the integrated region was also statistically significant (OR:1.60; 95% CI: 1.09–2.37), but we were unable to determine what was driving this association.

For *IGFBP3*, a large region across the gene was considered noteworthy using a sliding window analysis (Supplementary Figure S3). Two regions were defined by evaluating linkage disequilibrium across the identified area. The second region, spanning the area of exon 5 to 3′ downstream of *IGFBP3*, was associated with a global p-value of 0.04. Among haplotypes in this region, variant haplotype AGC (OR: 1.27, 95% CI: 1.06–1.54) and TAT (OR: 1.62, 95% CI: 1.05–2.51) were associated with increased renal cancer risk (Table 3). Among SNPs in the haplotype, rs6670 was statistically significantly associated with renal cancer risk at a FDR level <20%. We observed a positive association between renal cancer risk among subjects that had at least one copy of the rare allele with an OR of 1.27 (95% CI: 1.04–1.56). The association for haplotype AGC, which contains the rare allele for rs6670, was slightly stronger than the effect observed for the individual SNP and appears to be driven primarily by rs6670. The causal variant for haplotype TAT, however, is not apparent, suggesting that the causal variant was not genotyped in this study.

In *VCAM1*, a variant haplotype centered on rs3917010 was also associated with an increased risk of renal cancer (CAT OR: 1.25, 95% CI: 1.01–1.54; Table 3). However, none of the *VCAM1* SNPs were significantly associated with renal cancer risk after FDR adjustment. Although a statistically significant association was observed, this association could be spurious as the effects observed for the haplotype are not concordant with the individual SNP associations within this haplotype (Supplementary Table S1).

Results for individual analyses of all SNPs can be found in Supplemental Table S1. No statistically significant interactions between our statistically significant SNPs and potential effect modifiers (age, sex, and BMI) were detected (data not shown). Additional sensitivity analyses restricted to RCC (n = 627 cases) and clear cell RCC (n = 564 cases) did not meaningfully change any of the previously detected associations (data not shown).

## Discussion

In this study, we conducted an exploratory analysis of 319 SNPs in or around 21 genes involved in cell growth/differentiation and apoptosis pathways in relation to renal cancer risk. We identified both haplotypes and SNPs in *CASP1/5/4/12*, *EGFR*, and *IGFBP3* that were statistically significantly associated with risk of renal cancer. Associations between SNPs in the other investigated cell growth/differentiation and apoptosis pathway genes were weak and less promising.

There is strong evidence supporting the biological relevance of genetic variants in *EGFR* and *IGFBP3* and renal cancer risk. *EGFR* encodes for a transmembrane growth factor receptor that plays a critical role in the signal transduction pathway regulating cell proliferation, differentiation, and survival [17], [18]. A recent study has proposed an additional role for EGFR of interacting with and stabilizing the sodium/glucose cotransporter 1 (SGLT1), thus helping to maintain intracellular glucose levels in low extracellular glucose environments and prevent cell death from occurring [19]. This is especially relevant to renal cancer, as both EGFR and SGLT1 are expressed in the kidney, where glucose uptake is important [20]. Altered glucose metabolism is one of the major hypotheses thought to explain the association between diabetes and renal cancer. Thus far, most studies have focused on evaluating *EGFR* in relation to cancer progression and targeted treatment [21], [22]. It is interesting to note that the first intron of *EGFR* (>120 kb) has been implicated as an important regulatory area [21], [23]. A highly polymorphic (CA)_n_ repeat in intron 1 of *EGFR*, about 1.5 kb downstream of exon 1, has been associated with decreased *EGFR* transcription in multiple studies [24], [25]. This microsatellite appears to be in linkage disequilibrium with several SNPs of unknown function in the promoter region of this gene, as well[26]. One of these variants (rs759171) was also genotyped in this study, but not associated with renal cancer risk (Supplementary Table S1). In this study, three SNPs (rs11238349, rs6954351, and rs7796139) from intron 1 of *EGFR* and identified through our initial screen were statistically significantly associated with risk of renal cancer. Among these three SNPs, only rs6954351 and rs7796139 were moderately correlated (r^2^ = 0.47) with one another. Subsequent analyses suggest that perhaps a haplotype that includes these two SNPs may be driving the associations found in this region. The mechanism through which these intronic SNPs (or variants in linkage disequilibrium with these SNPs) might affect renal cancer risk is unknown but they do reside within a functionally relevant region of *EGFR* that has been associated with decreased EGFR transcription and protein expression in humans.

Similar to our findings for *EGFR*, the *IGFBP3* regions associated with modified risk appear to be functionally important in cancer. *IGFBP3* encodes for IGF-binding protein 3 and is the primary carrier of circulating IGF-1. A reduction in the amount of IGFBP3 available results in an increase in levels of free IGF-1, a factor associated with growth, proliferation, and an elevated risk of several cancers [27], [28]. Independent of IGF-1, IGFBP3 has also been shown to affect cell proliferation and apoptosis through its interactions with several signaling pathways [29], [30]. In relation to renal cancer, experimental studies have demonstrated that *IGFBP3* expression is increased among both clear cell renal tumors and renal cancer cell lines [31], [32]. The promoter region of *IGFBP3* has also been observed to be frequently hypermethylated in primary renal cell tumors, but unmethhylated among normal cells [33]. We observed a statistically significant increase in renal cancer risk with rs6670 located in the 3′ untranslated region (UTR) of *IGFBP3*. Variants in the 3′UTR could be involved in the stability and expression of mRNA [34]. *IGFBP3* variation has been evaluated with several other cancer sites [35], but this is the first study to evaluate SNPs in relation to renal cancer. In association studies, SNPs in *IGFBP3* and IGF related genes (*IGF-1* and *IGFBP1*) have been related to circulating IGF-1 and IGFBP-3 levels [36], [37]. *IGFBP3* SNP rs6670 (A allele) was not directly associated with IGFBP-3 levels but was weakly associated with a decreasing trend in circulating IGF-1 levels [36]. This is not entirely consistent with the positive association we observed with renal cancer in our study, but suggests that further study is needed to clarify the associations observed.

*CASP1*, *CASP4*, *CASP5* and *CASP12* belong to a caspase subfamily called the inflammatory caspases, which are involved in the maturation of inflammatory cytokines (Il-1β and IL-18) in addition to their role in apoptotic pathways [9], [38], [39]. Despite their involvement in two key carcinogenic pathways, inflammation and apoptosis, few published reports have evaluated genetic variation in these four caspase genes in relation to cancer. In our study, three *CASP1/5/4/12* SNPs (rs568910, rs492859, rs507879) were associated with an increased risk of renal cancer, while one SNP (rs1785883) was associated with a decreased risk. The four SNPs were only weakly correlated with each other (r^2^<0.5), except for rs492859 and rs568910 which were strongly correlated (r^2^ = 0.99) within our data. The strongest individual SNP association with renal cancer was observed with rs507879, located within exon 2 of *CASP5* and results in a missense mutation and amino acid substitution (Thr90Ala). The function of this particular exon 2 SNP is unclear and is predicted to be a benign mutation by PolyPhen. However, a common somatic mutation in exon 2 has also been identified in leukemias and, gastric, colon, and lung cancers, but has not yet been examined in renal tumors [40]–[43]. A somatic mutation in a mononucleotide repeat (A)_10_ in exon 2 produces a shift in the reading frame during transcription resulting in a premature stop and a truncated protein. This suggests that this region in *CASP5* may be particularly important for carcinogenesis.

To our knowledge, this is the first study to evaluate SNPs in all but two of these growth/differentiation and apoptosis genes in relation to renal cancer. The primary focus so far in the area of renal cancer susceptibility has been on genetic variants in xenobiotic metabolism genes [12], [44] and the von Hippel-Lindau (*VHL*) gene, which leads to an increased risk of the hereditary form of renal cancer [45], Only three small studies have evaluated variants in *PPARG* and *VEGF* in relation to renal cancer. Smith et al. (n = 40 cases) observed that the rare allele of the *PPARG* P12A polymorphism (rs1801282) was underrepresented among RCC patients compared to controls, with an OR for trend of 0.28 (0.08–1.01) [46]. This finding is consistent with results from our analysis (OR for trend: 0.80; 95% CI: 0.67–0.96), but this SNP was not considered statistically significant after FDR adjustment. Kawai et al. (n = 213 cases) [47] observed a weak association between three *VEGF* promoter polymorphisms (rs1570360, rs2010963, rs699947) and renal cancer progression and prognosis; and Abe et al. (n = 145 cases) [48] observed a nonsignificant association between three *VEGF* 3′UTR polymorphisms (C702T −dbSNP identifier number is unknown, rs3025039, rs10434) and renal cancer risk in Japanese populations. Three of these SNPs were genotyped in our study (rs2010963, rs699947, rs3025039), but only SNP rs699947 demonstrated a weak but nonsignificant association with renal cancer risk. Our analysis of *VEGF* revealed only one nominally significant SNP in the promoter region (rs833058; Supplemental Table S1) which is correlated with rs699947 (r^2^ = 0.65).

A strength of our study is the large sample size which provides sufficient statistical power to detect associations between SNPs and renal cancer risk. Hospital-based controls in our study could potentially cause selection bias if carrying specific genetic variants were somehow related to hospitalization or if the controls were somehow not representative of the general population. However, the high participation and response rates among both cases and controls minimize the potential for selection bias. Given the multiple centers and countries in our study, the potential for population stratification exists; however, we found no evidence of heterogeneity. Population stratification may still be present, but the likelihood of this is small among European populations [49]. Although tagSNP selection was not based on resequencing data, the strategy for selecting tagSNPs allowed a more comprehensive analysis of common genetic variation in these genes than the traditional candidate SNP approach. Given the large number of associations investigated, additional examination of statistically significant associations using FDR control helped us to evaluate the potential for chance findings due to multiple testing. Results from the replication conducted within the US Kidney Cancer study for rs507879 were not statistically significant on their own, but the study (696 cases and 593 controls) was underpowered (40%) to detect an association of 1.3. Point estimates calculated by pooling data from the two studies may better represent the true association between rs507879 and renal cancer.

In summary, the results from this study suggest that genetic polymorphisms and haplotypes within the *CASP1*, *CASP5*, *EGFR*, and *IGFBP3* genes are associated with renal cancer risk. The regions identified in this study appear to have functional relevance in renal and other types of cancer. To our knowledge, this is one of the largest evaluations of genetic susceptibility and renal cancer conducted to date, but there is need to replicate and extend our findings in other populations.

## Supporting Information

Figure S1 Sliding window results and linkage disequilibrium plot of CASP1/5/4/12 region. SNPs associated or located within a CASP gene are indicated by their respective lines. The haplotype results reported in Table 3 are indicated by a line depicting Region1. Upper portion of figure presents global p-value associated with each 3 SNP sliding window, unadjusted and FDR-adjusted. Lower portion of figure presents linkage disequilibrium plot with color scheme based on D' and logarithm of the odds of linkage (LOD) scores. Numbers in the squares are r2 values.
(4.14 MB TIF)

Figure S2 Sliding window results and linkage disequilibrium plot of EGFR region. The haplotype results for EGFR reported in Table 3 are indicated by lines depicting each region. Upper portion of figure presents global p-value associated with each 3 SNP sliding window, unadjusted and FDR-adjusted. Lower portion of figure presents linkage disequilibrium plot with color scheme based on D' and logarithm of the odds of linkage (LOD) scores. Numbers in the squares are r2 values.
(5.86 MB TIF)

Figure S3 Sliding window results and linkage disequilibrium plot of IGFBP3 region. The haplotype results reported in Table 3 are indicated by a line depicting each region. Upper portion of figure presents global p-value associated with each 3 SNP sliding window, unadjusted and FDR-adjusted. Lower portion of figure presents linkage disequilibrium plot with color scheme based on D' and logarithm of the odds of linkage (LOD) scores. Numbers in the squares are r2 values.
(3.22 MB TIF)

Table S1 Results from all Growth and Differentiation, Apoptosis Polymorphisms and Renal Cell Cancer Risk.
(2.66 MB DOC)

Table S2 EGFR integrated haplotype and renal cancer risk.
(0.06 MB DOC)
