# Supplementary figures and images for: An Analysis of Growth, Differentiation and Apoptosis Genes with Risk of Renal Cancer
Source: PLoS One. 2009 Mar 24;4(3):e4895. doi: 10.1371/journal.pone.0004895 (PMC2656573; doi:10.1371/journal.pone.0004895)

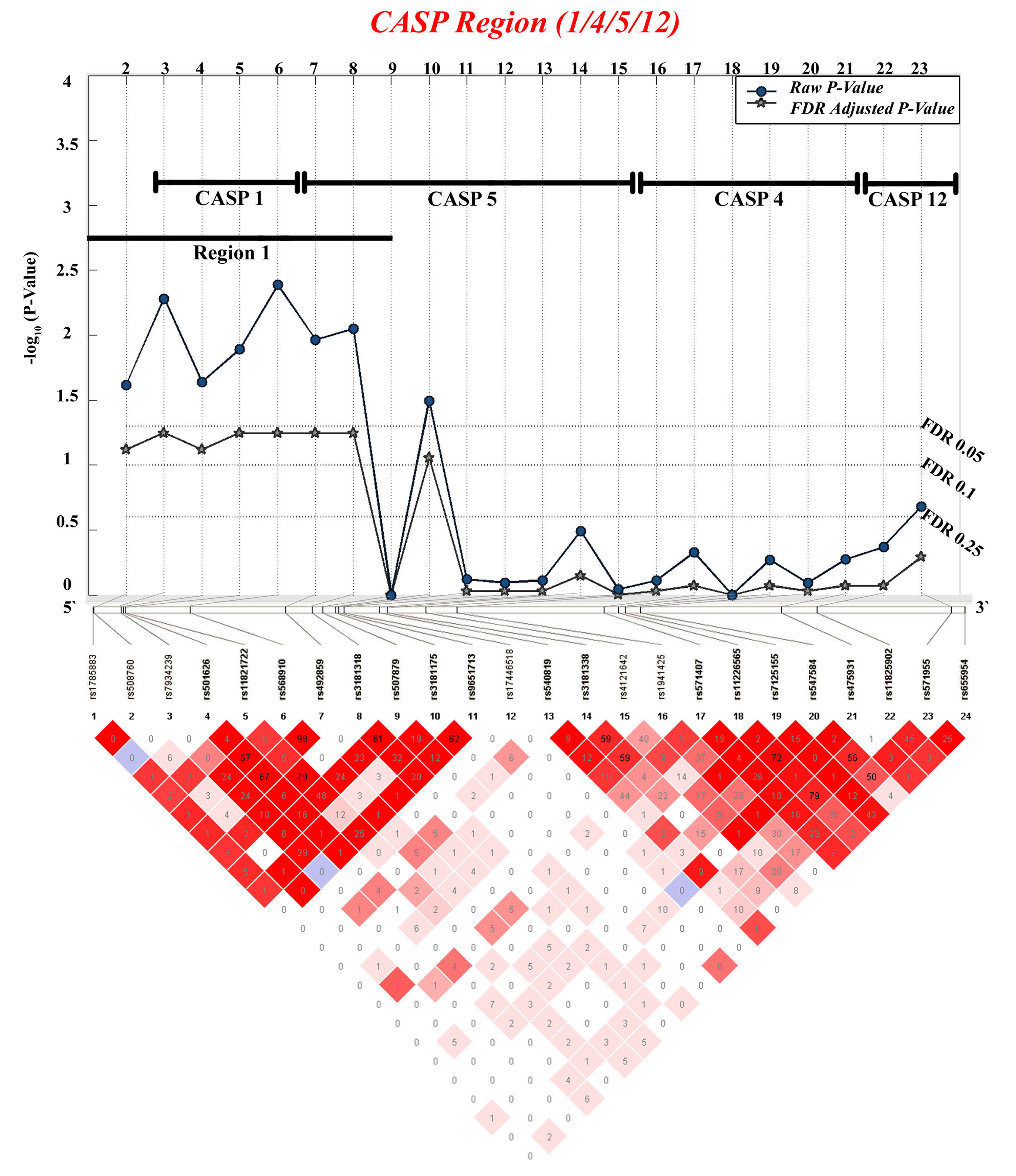

Supplement: Figure S1 — Sliding window results and linkage disequilibrium plot of CASP1/5/4/12 region. SNPs associated or located within a CASP gene are indicated by their respective lines. The haplotype results reported in Table 3 are indicated by a line depicting Region1. Upper portion of figure presents global p-value associated with each 3 SNP sliding window, unadjusted and FDR-adjusted. Lower portion of figure presents linkage disequilibrium plot with color scheme based on D' and logarithm of the odds of linkage (LOD) scores. Numbers in the squares are r2 values. (4.14 MB TIF) [file pone.0004895.s001.tif]

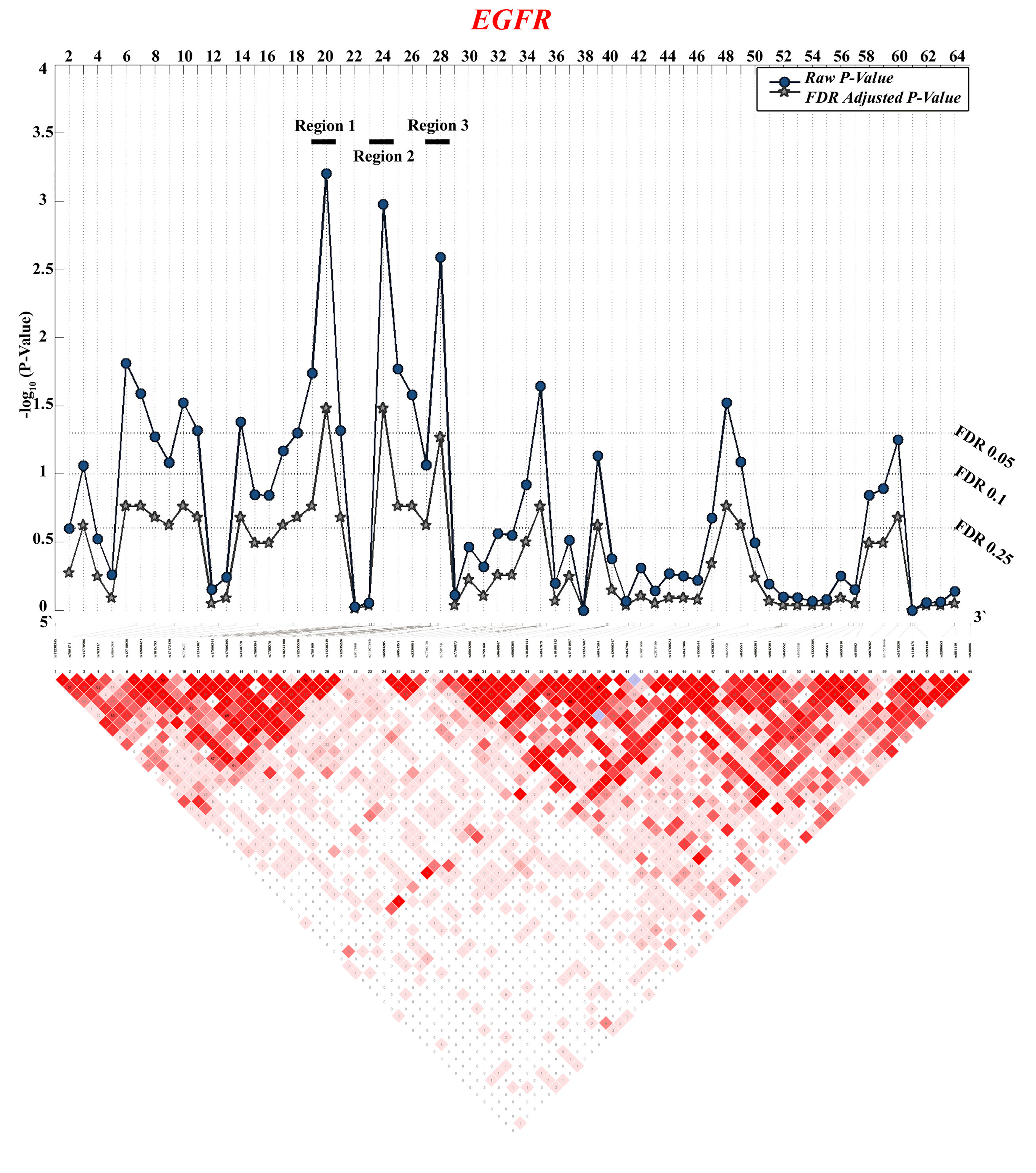

Supplement: Figure S2 — Sliding window results and linkage disequilibrium plot of EGFR region. The haplotype results for EGFR reported in Table 3 are indicated by lines depicting each region. Upper portion of figure presents global p-value associated with each 3 SNP sliding window, unadjusted and FDR-adjusted. Lower portion of figure presents linkage disequilibrium plot with color scheme based on D' and logarithm of the odds of linkage (LOD) scores. Numbers in the squares are r2 values. (5.86 MB TIF) [file pone.0004895.s002.tif]

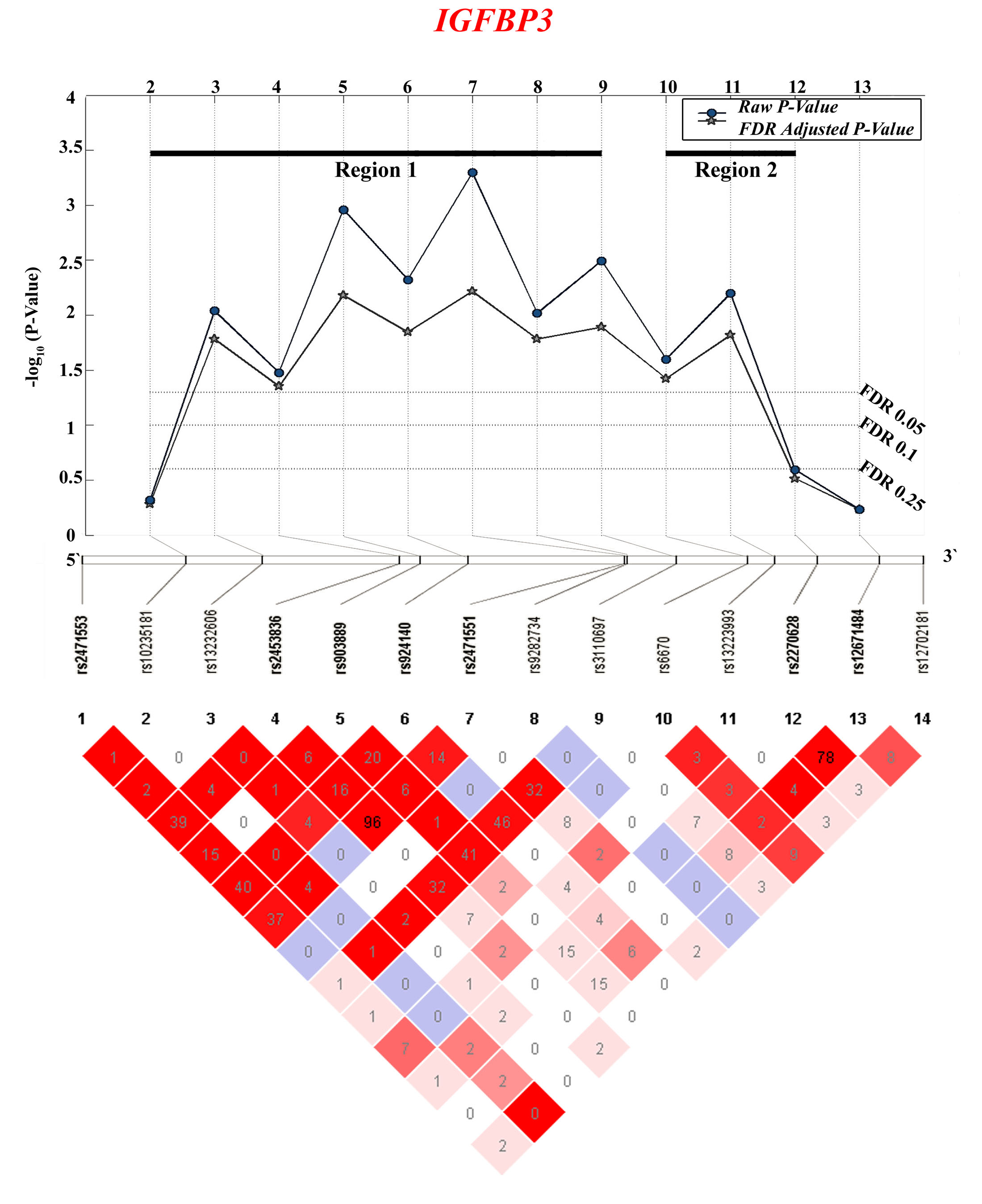

Supplement: Figure S3 — Sliding window results and linkage disequilibrium plot of IGFBP3 region. The haplotype results reported in Table 3 are indicated by a line depicting each region. Upper portion of figure presents global p-value associated with each 3 SNP sliding window, unadjusted and FDR-adjusted. Lower portion of figure presents linkage disequilibrium plot with color scheme based on D' and logarithm of the odds of linkage (LOD) scores. Numbers in the squares are r2 values. (3.22 MB TIF) [file pone.0004895.s003.tif]
