# Supplementary material for: An Analysis of Growth, Differentiation and Apoptosis Genes with Risk of Renal Cancer
Source: PLoS One. 2009 Mar 24;4(3):e4895. doi: 10.1371/journal.pone.0004895 (PMC2656573; doi:10.1371/journal.pone.0004895)
Supplement: Table S1 — Results from all Growth and Differentiation, Apoptosis Polymorphisms and Renal Cell Cancer Risk. (2.66 MB DOC) [file pone.0004895.s004.doc]

**Supplementary Table 1. Growth and Differentiation, Apoptosis** Polymorphisms and Renal Cell Cancer Risk

| **SNP/Genotypes** | **Cases** | **Controls** | **OR^1^** | **95% CI** | | **p-trend** |
| --- | --- | --- | --- | --- | --- | --- |
| ***AKR1C3*** |  |  |  |  |  |  |
| rs6601899 (-18314A>C) |  |  |  |  |  |  |
| CC | 542 | 755 | 1.00 |  |  |  |
| AC | 211 | 249 | 1.18 | 0.95 | 1.47 |  |
| AA | 19 | 26 | 1.02 | 0.56 | 1.87 | 0.21 |
| AC+AA |  |  | 1.17 | 0.95 | 1.44 |  |
| rs10752001 (-17791C>T) |  |  |  |  |  |  |
| TT | 547 | 757 | 1.00 |  |  |  |
| CT | 210 | 249 | 1.17 | 0.94 | 1.45 |  |
| CC | 19 | 26 | 1.01 | 0.55 | 1.86 | 0.25 |
| CT+CC |  |  | 1.15 | 0.93 | 1.41 |  |
| rs11252920 (-17543A>C) |  |  |  |  |  |  |
| CC | 373 | 475 | 1.00 |  |  |  |
| AC | 339 | 454 | 0.96 | 0.79 | 1.17 |  |
| AA | 65 | 104 | 0.79 | 0.56 | 1.11 | 0.24 |
| AC+AA |  |  | 0.92 | 0.77 | 1.11 |  |
| rs11597709 (-14210A>G) |  |  |  |  |  |  |
| GG | 681 | 910 | 1.00 |  |  |  |
| AG | 89 | 120 | 0.98 | 0.73 | 1.32 |  |
| AA | 5 | 2 | 3.05 | 0.59 | 15.81 | 0.72 |
| AG+AA |  |  | 1.01 | 0.76 | 1.34 |  |
| rs6601900 (-13500A>G) |  |  |  |  |  |  |
| GG | 261 | 324 | 1.00 |  |  |  |
| AG | 360 | 498 | 0.92 | 0.74 | 1.14 |  |
| AA | 156 | 212 | 0.92 | 0.70 | 1.19 | 0.47 |
| AG+AA |  |  | 0.92 | 0.75 | 1.12 |  |
| rs10795243 (-13367C>G) |  |  |  |  |  |  |
| GG | 446 | 626 | 1.00 |  |  |  |
| CG | 283 | 365 | 1.10 | 0.90 | 1.34 |  |
| CC | 48 | 43 | **1.59** | **1.03** | **2.45** | 0.06 |
| CG+CC |  |  | 1.15 | 0.95 | 1.39 |  |
| rs6601901 (-13129T>G) |  |  |  |  |  |  |
| TT | 350 | 458 | 1.00 |  |  |  |
| GT | 338 | 447 | 0.98 | 0.81 | 1.20 |  |
| GG | 83 | 125 | 0.84 | 0.62 | 1.15 | 0.37 |
| GT+GG |  |  | 0.96 | 0.79 | 1.15 |  |
| rs12529 (Ex1-70C>G) |  |  |  |  |  |  |
| CC | 257 | 348 | 1.00 |  |  |  |
| CG | 398 | 504 | 1.08 | 0.87 | 1.33 |  |
| GG | 122 | 182 | 0.93 | 0.70 | 1.23 | 0.79 |
| CG+GG |  |  | 1.03 | 0.85 | 1.26 |  |
| rs17396032 (IVS1-756A>G) |  |  |  |  |  |  |
| AA | 688 | 907 | 1.00 |  |  |  |
| AG | 79 | 116 | 0.90 | 0.66 | 1.22 |  |
| GG | 1 | 2 | 0.74 | 0.07 | 8.44 | 0.46 |
| AG+GG |  |  | 0.91 | 0.68 | 1.22 |  |
| rs2245191 (IVS3+73C>A) |  |  |  |  |  |  |
| CC | 504 | 611 | 1.00 |  |  |  |
| AC | 277 | 369 | 0.90 | 0.74 | 1.10 |  |
| AA | 35 | 60 | 0.69 | 0.45 | 1.07 | 0.08 |
| AC+AA |  |  | 0.89 | 0.75 | 1.06 |  |
| rs1937841 (IVS4+218G>A) |  |  |  |  |  |  |
| GG | 619 | 839 | 1.00 |  |  |  |
| AG | 144 | 183 | 1.07 | 0.84 | 1.36 |  |
| AA | 9 | 11 | 1.18 | 0.48 | 2.91 | 0.54 |
| AG+AA |  |  | 1.10 | 0.87 | 1.39 |  |
| rs4881400 (IVS5-256T>G) |  |  |  |  |  |  |
| TT | 394 | 554 | 1.00 |  |  |  |
| GT | 320 | 416 | 1.09 | 0.89 | 1.32 |  |
| GG | 63 | 64 | **1.45** | **1.00** | **2.11** | 0.08 |
| GT+GG |  |  | 1.13 | 0.94 | 1.37 |  |
| rs3750566 (IVS6+121A>G) |  |  |  |  |  |  |
| AA | 503 | 696 | 1.00 |  |  |  |
| AG | 252 | 306 | 1.15 | 0.93 | 1.40 |  |
| GG | 22 | 33 | 0.97 | 0.56 | 1.69 | 0.34 |
| AG+GG |  |  | 1.13 | 0.93 | 1.38 |  |
| rs4242785 (IVS8-509A>G) |  |  |  |  |  |  |
| GG | 420 | 622 | 1.00 |  |  |  |
| AG | 317 | 353 | **1.35** | **1.11** | **1.65** |  |
| AA | 39 | 59 | 0.96 | 0.63 | 1.48 | **0.05** |
| AG+AA |  |  | **1.30** | **1.07** | **1.57** |  |
| rs7894034 (656bp 3' of STP G>T) |  |  |  |  |  |  |
| GG | 697 | 943 | 1.00 |  |  |  |
| GT | 76 | 87 | 1.19 | 0.86 | 1.64 |  |
| TT | 3 | 4 | 0.91 | 0.20 | 4.08 | 0.37 |
| GT+TT |  |  | 1.17 | 0.85 | 1.61 |  |
| rs7086771 (Ex9+1940C>G) |  |  |  |  |  |  |
| GG | 320 | 481 | 1.00 |  |  |  |
| CG | 363 | 432 | **1.27** | **1.04** | **1.55** |  |
| CC | 94 | 121 | 1.20 | 0.88 | 1.63 | 0.06 |
| CG+CC |  |  | **1.25** | **1.04** | **1.51** |  |
| rs7068685 (*47054T>C) |  |  |  |  |  |  |
| AA | 466 | 596 | 1.00 |  |  |  |
| AG | 279 | 380 | 0.94 | 0.78 | 1.15 |  |
| GG | 32 | 57 | 0.71 | 0.45 | 1.11 | 0.19 |
| AG+GG |  |  | 0.91 | 0.75 | 1.10 |  |
|  |  |  |  |  |  |  |
| ***EGF*** |  |  |  |  |  |  |
| rs2347134 (-20034A>T) |  |  |  |  |  |  |
| TT | 460 | 644 | 1.00 |  |  |  |
| AT | 274 | 340 | 1.15 | 0.94 | 1.41 |  |
| AA | 43 | 50 | 1.24 | 0.81 | 1.91 | 0.1168 |
| AT+AA |  |  | 1.16 | 0.96 | 1.41 |  |
| rs718768 (-10376G>A) |  |  |  |  |  |  |
| AA | 396 | 551 | 1.00 |  |  |  |
| AG | 315 | 414 | 1.06 | 0.87 | 1.30 |  |
| GG | 62 | 64 | 1.36 | 0.93 | 1.98 | 0.1533 |
| AG+GG |  |  | 1.10 | 0.91 | 1.33 |  |
| rs17316633 (-8857G>A) |  |  |  |  |  |  |
| GG | 460 | 610 | 1.00 |  |  |  |
| AG | 269 | 372 | 0.97 | 0.79 | 1.19 |  |
| AA | 47 | 52 | 1.22 | 0.80 | 1.85 | 0.6985 |
| AG+AA |  |  | 1.00 | 0.83 | 1.21 |  |
| rs3756261( -2185 T>C) |  |  |  |  |  |  |
| TT | 667 | 860 | 1.00 |  |  |  |
| CT | 98 | 155 | 0.83 | 0.63 | 1.09 |  |
| CC | 7 | 10 | 0.99 | 0.37 | 2.65 | 0.2369 |
| CT+CC |  |  | 0.82 | 0.64 | 1.07 |  |
| rs6823529 (IVS1-12594G>C) |  |  |  |  |  |  |
| GG | 643 | 832 | 1.00 |  |  |  |
| CG | 115 | 173 | 0.86 | 0.67 | 1.12 |  |
| CC | 7 | 13 | 0.74 | 0.29 | 1.88 | 0.2086 |
| CG+CC |  |  | 0.86 | 0.67 | 1.09 |  |
| rs1860129 (IVS10+650G>C) |  |  |  |  |  |  |
| GG | 275 | 356 | 1.00 |  |  |  |
| CG | 379 | 510 | 0.96 | 0.78 | 1.19 |  |
| CC | 121 | 169 | 0.96 | 0.72 | 1.27 | 0.7117 |
| CG+CC |  |  | 0.97 | 0.79 | 1.18 |  |
| rs2255355 (IVS11+1268A>C) |  |  |  |  |  |  |
| AA | 654 | 850 | 1.00 |  |  |  |
| AC | 113 | 171 | 0.86 | 0.67 | 1.12 |  |
| CC | 10 | 14 | 0.97 | 0.43 | 2.22 | 0.3314 |
| AC+CC |  |  | 0.87 | 0.68 | 1.12 |  |
| rs7670908 (IVS11+2350G>A) |  |  |  |  |  |  |
| GG | 658 | 843 | 1.00 |  |  |  |
| AG | 111 | 175 | 0.82 | 0.63 | 1.06 |  |
| AA | 8 | 13 | 0.83 | 0.34 | 2.03 | 0.1357 |
| AG+AA |  |  | 0.80 | 0.62 | 1.03 |  |
| rs11568993 (Ex13-77C>T) |  |  |  |  |  |  |
| CC | 674 | 904 | 1.00 |  |  |  |
| CT | 98 | 127 | 1.00 | 0.76 | 1.33 |  |
| TT | 4 | 4 | 1.25 | 0.31 | 5.05 | 0.8889 |
| CT+TT |  |  | 1.03 | 0.78 | 1.36 |  |
| rs11568994 (IVS13+144G>A) |  |  |  |  |  |  |
| GG | 369 | 491 | 1.00 |  |  |  |
| AG | 330 | 447 | 0.99 | 0.81 | 1.21 |  |
| AA | 78 | 96 | 1.11 | 0.80 | 1.55 | 0.7019 |
| AG+AA |  |  | 1.01 | 0.84 | 1.22 |  |
| rs7692976 (IVS18+1702A>G) |  |  |  |  |  |  |
| AA | 243 | 291 | 1.00 |  |  |  |
| AG | 388 | 530 | 0.88 | 0.71 | 1.09 |  |
| GG | 146 | 213 | 0.84 | 0.64 | 1.11 | 0.1888 |
| AG+GG |  |  | 0.87 | 0.71 | 1.06 |  |
| rs4698803 (Ex19+25A>T ) |  |  |  |  |  |  |
| TT | 494 | 655 | 1.00 |  |  |  |
| AT | 248 | 344 | 0.94 | 0.77 | 1.15 |  |
| AA | 35 | 36 | 1.27 | 0.78 | 2.06 | 0.9049 |
| AT+AA |  |  | 0.97 | 0.80 | 1.18 |  |
| rs2299001 (IVS20+1451T>C ) |  |  |  |  |  |  |
| TT | 654 | 856 | 1.00 |  |  |  |
| CT | 108 | 162 | 0.88 | 0.68 | 1.15 |  |
| CC | 7 | 11 | 0.90 | 0.35 | 2.36 | 0.3716 |
| CT+CC |  |  | 0.91 | 0.71 | 1.17 |  |
| rs6533485 (IVS22-1745G>C ) |  |  |  |  |  |  |
| GG | 214 | 266 | 1.00 |  |  |  |
| CG | 383 | 523 | 0.91 | 0.73 | 1.14 |  |
| CC | 180 | 246 | 0.92 | 0.71 | 1.21 | 0.54 |
| CG+CC |  |  | 0.92 | 0.74 | 1.13 |  |
| rs17041230 (4903bp 3' of STP C>T ) |  |  |  |  |  |  |
| CC | 632 | 828 | 1.00 |  |  |  |
| CT | 125 | 188 | 0.87 | 0.68 | 1.12 |  |
| TT | 10 | 12 | 1.15 | 0.49 | 2.70 | 0.4386 |
| CT+TT |  |  | 0.92 | 0.73 | 1.17 |  |
| EGF_22, 9984bp 3' of STP A>G (rs10021804 ) |  |  |  |  |  |  |
| AA | 289 | 381 | 1.00 |  |  |  |
| AG | 372 | 495 | 0.99 | 0.81 | 1.22 |  |
| GG | 115 | 158 | 1.00 | 0.75 | 1.33 | 0.9663 |
| AG+GG |  |  | 0.99 | 0.82 | 1.21 |  |
|  |  |  |  |  |  |  |
| ***EGFR*** |  |  |  |  |  |  |
| rs11238345 (-19498G>A) |  |  |  |  |  |  |
| GG | 434 | 571 | 1.00 |  |  |  |
| AG | 307 | 395 | 1.02 | 0.84 | 1.24 |  |
| AA | 33 | 67 | 0.68 | 0.44 | 1.05 | 0.3574 |
| AG+AA |  |  | 0.98 | 0.81 | 1.18 |  |
| rs759171 (-758A>C) |  |  |  |  |  |  |
| CC | 539 | 725 | 1.00 |  |  |  |
| AC | 219 | 276 | 1.08 | 0.87 | 1.33 |  |
| AA | 19 | 28 | 0.98 | 0.54 | 1.78 | 0.6073 |
| AC+AA |  |  | 1.05 | 0.85 | 1.28 |  |
| rs11770506 (IVS1+3321T>C) |  |  |  |  |  |  |
| TT | 365 | 469 | 1.00 |  |  |  |
| CT | 354 | 460 | 0.98 | 0.81 | 1.19 |  |
| CC | 57 | 104 | 0.73 | 0.51 | 1.04 | 0.1834 |
| CT+CC |  |  | 0.93 | 0.77 | 1.13 |  |
| rs763317 (IVS1+8139A>G) |  |  |  |  |  |  |
| GG | 179 | 284 | 1.00 |  |  |  |
| AG | 405 | 507 | 1.22 | 0.97 | 1.54 |  |
| AA | 192 | 243 | 1.23 | 0.94 | 1.61 | 0.1297 |
| AG+AA |  |  | 1.22 | 0.98 | 1.52 |  |
| rs6956366 (IVS1+14443G>C) |  |  |  |  |  |  |
| GG | 372 | 461 | 1.00 |  |  |  |
| CG | 343 | 458 | 0.92 | 0.76 | 1.12 |  |
| CC | 62 | 115 | **0.69** | **0.49** | **0.96** | **0.0465** |
| CG+CC |  |  | 0.87 | 0.72 | 1.05 |  |
| rs12718939 (IVS1+18262A>G) |  |  |  |  |  |  |
| GG | 336 | 492 | 1.00 |  |  |  |
| AG | 372 | 424 | **1.29** | **1.06** | **1.57** |  |
| AA | 68 | 118 | 0.86 | 0.62 | 1.20 | 0.5405 |
| AG+AA |  |  | 1.20 | 0.99 | 1.45 |  |
| rs12668421 (IVS1+22119A>T) |  |  |  |  |  |  |
| AA | 478 | 586 | 1.00 |  |  |  |
| AT | 267 | 380 | 0.85 | 0.70 | 1.04 |  |
| TT | 32 | 69 | **0.57** | **0.37** | **0.89** | **0.0079** |
| AT+TT |  |  | **0.81** | **0.67** | **0.98** |  |
| rs1015793 (IVS1+27258A>G) |  |  |  |  |  |  |
| AA | 539 | 719 | 1.00 |  |  |  |
| AG | 220 | 288 | 1.03 | 0.84 | 1.27 |  |
| GG | 18 | 28 | 0.90 | 0.49 | 1.66 | 0.9406 |
| AG+GG |  |  | 1.02 | 0.83 | 1.25 |  |
| rs17172430 (IVS1+35592G>A) |  |  |  |  |  |  |
| GG | 612 | 814 | 1.00 |  |  |  |
| AG | 152 | 211 | 0.98 | 0.77 | 1.23 |  |
| AA | 10 | 9 | 1.56 | 0.63 | 3.90 | 0.8299 |
| AG+AA |  |  | 1.01 | 0.80 | 1.27 |  |
| rs723527 (IVS1+47814A>G) |  |  |  |  |  |  |
| AA | 295 | 358 | 1.00 |  |  |  |
| AG | 374 | 489 | 0.92 | 0.75 | 1.14 |  |
| GG | 105 | 187 | **0.69** | **0.52** | **0.92** | **0.0202** |
| AG+GG |  |  | 0.87 | 0.71 | 1.05 |  |
| rs1344307 (IVS1+50830G>A) |  |  |  |  |  |  |
| AA | 450 | 605 | 1.00 |  |  |  |
| AG | 292 | 361 | 1.08 | 0.89 | 1.32 |  |
| GG | 35 | 68 | 0.69 | 0.45 | 1.06 | 0.5975 |
| AG+GG |  |  | 1.02 | 0.84 | 1.23 |  |
| rs17586344 (IVS1+53340T>C) |  |  |  |  |  |  |
| TT | 575 | 785 | 1.00 |  |  |  |
| CT | 187 | 237 | 1.07 | 0.86 | 1.34 |  |
| CC | 14 | 12 | 1.72 | 0.78 | 3.78 | 0.2579 |
| CT+CC |  |  | 1.10 | 0.89 | 1.37 |  |
| rs17586365 (IVS1+53728G>A) |  |  |  |  |  |  |
| GG | 571 | 758 | 1.00 |  |  |  |
| AG | 194 | 248 | 1.06 | 0.85 | 1.32 |  |
| AA | 12 | 28 | 0.57 | 0.29 | 1.13 | 0.6788 |
| AG+AA |  |  | 1.01 | 0.81 | 1.24 |  |
| rs4140770 (IVS1+54418G>A) |  |  |  |  |  |  |
| AA | 297 | 401 | 1.00 |  |  |  |
| AG | 377 | 463 | 1.12 | 0.91 | 1.37 |  |
| GG | 103 | 171 | 0.82 | 0.61 | 1.09 | 0.4597 |
| AG+GG |  |  | 1.04 | 0.85 | 1.26 |  |
| rs7809394 (IVS1+58324C>T) |  |  |  |  |  |  |
| CC | 490 | 711 | 1.00 |  |  |  |
| CT | 260 | 290 | **1.29** | **1.05** | **1.59** |  |
| TT | 26 | 32 | 1.13 | 0.66 | 1.93 | **0.0321** |
| CT+TT |  |  | **1.27** | **1.05** | **1.55** |  |
| rs7780270 (IVS1-58093G>T) |  |  |  |  |  |  |
| GG | 252 | 309 | 1.00 |  |  |  |
| GT | 364 | 479 | 0.94 | 0.75 | 1.16 |  |
| TT | 161 | 247 | 0.81 | 0.62 | 1.05 | 0.1222 |
| GT+TT |  |  | 0.89 | 0.73 | 1.09 |  |
| rs10244108 (IVS1-57642G>A) |  |  |  |  |  |  |
| GG | 350 | 526 | 1.00 |  |  |  |
| AG | 336 | 409 | **1.23** | **1.01** | **1.50** |  |
| AA | 90 | 100 | 1.33 | 0.97 | 1.83 | **0.0224** |
| AG+AA |  |  | **1.25** | **1.04** | **1.51** |  |
| rs12535536 (IVS1-55598G>A) |  |  |  |  |  |  |
| AA | 334 | 457 | 1.00 |  |  |  |
| AG | 361 | 446 | 1.11 | 0.91 | 1.36 |  |
| GG | 79 | 124 | 0.88 | 0.64 | 1.21 | 0.9211 |
| AG+GG |  |  | 1.06 | 0.87 | 1.27 |  |
| rs759169 (IVS1-55343T>C) |  |  |  |  |  |  |
| CC | 487 | 650 | 1.00 |  |  |  |
| CT | 261 | 324 | 1.08 | 0.88 | 1.32 |  |
| TT | 25 | 57 | **0.59** | **0.36** | **0.96** | 0.4454 |
| CT+TT |  |  | 1.01 | 0.83 | 1.23 |  |
| rs11238349 (IVS1-53908G>A) |  |  |  |  |  |  |
| GG | 484 | 580 | 1.00 |  |  |  |
| AG | 248 | 398 | **0.75** | **0.61** | **0.91** |  |
| AA | 45 | 56 | 0.96 | 0.63 | 1.45 | **0.0412** |
| AG+AA |  |  | **0.77** | **0.64** | **0.93** |  |
| rs12535226 (IVS1-53560T>A) |  |  |  |  |  |  |
| TT | 248 | 339 | 1.00 |  |  |  |
| AT | 387 | 502 | 1.05 | 0.85 | 1.30 |  |
| AA | 141 | 191 | 0.98 | 0.75 | 1.30 | 0.9975 |
| AT+AA |  |  | 1.03 | 0.84 | 1.26 |  |
| rs917880 (IVS1-47968C>T) |  |  |  |  |  |  |
| CC | 230 | 298 | 1.00 |  |  |  |
| CT | 380 | 505 | 0.98 | 0.79 | 1.22 |  |
| TT | 167 | 232 | 0.93 | 0.71 | 1.21 | 0.59 |
| CT+TT |  |  | 0.97 | 0.79 | 1.19 |  |
| rs11977660 (IVS1-47643T>C) |  |  |  |  |  |  |
| TT | 220 | 285 | 1.00 |  |  |  |
| CT | 381 | 499 | 0.98 | 0.79 | 1.23 |  |
| CC | 176 | 251 | 0.88 | 0.68 | 1.15 | 0.3688 |
| CT+CC |  |  | 0.95 | 0.77 | 1.17 |  |
| rs6593205 (IVS1-41287A>G) |  |  |  |  |  |  |
| GG | 310 | 414 | 1.00 |  |  |  |
| AG | 368 | 456 | 1.09 | 0.89 | 1.34 |  |
| AA | 98 | 164 | 0.80 | 0.60 | 1.07 | 0.3731 |
| AG+AA |  |  | 1.02 | 0.84 | 1.23 |  |
| rs6954351 (IVS1-38789G>A) |  |  |  |  |  |  |
| GG | 522 | 752 | 1.00 |  |  |  |
| AG | 236 | 257 | **1.34** | **1.08** | **1.65** |  |
| AA | 18 | 26 | 1.04 | 0.56 | 1.93 | **0.0251** |
| AG+AA |  |  | **1.31** | **1.07** | **1.61** |  |
| rs2330951 (IVS1-35637C>A) |  |  |  |  |  |  |
| AA | 455 | 622 | 1.00 |  |  |  |
| AC | 278 | 338 | 1.13 | 0.92 | 1.38 |  |
| CC | 33 | 64 | 0.72 | 0.46 | 1.12 | 0.8915 |
| AC+CC |  |  | 1.07 | 0.88 | 1.29 |  |
| rs7796139 (IVS1-34103A>G) |  |  |  |  |  |  |
| AA | 370 | 543 | 1.00 |  |  |  |
| AG | 337 | 423 | 1.17 | 0.96 | 1.42 |  |
| GG | 70 | 68 | **1.52** | **1.06** | **2.18** | **0.0157** |
| AG+GG |  |  | **1.21** | **1.00** | **1.46** |  |
| rs759158 (IVS1-30770G>T) |  |  |  |  |  |  |
| TT | 253 | 351 | 1.00 |  |  |  |
| GT | 372 | 502 | 1.03 | 0.83 | 1.27 |  |
| GG | 151 | 181 | 1.16 | 0.88 | 1.52 | 0.3339 |
| GT+GG |  |  | 1.06 | 0.87 | 1.30 |  |
| rs7796872 (IVS1-30135G>A) |  |  |  |  |  |  |
| GG | 593 | 780 | 1.00 |  |  |  |
| AG | 170 | 236 | 0.94 | 0.75 | 1.18 |  |
| AA | 11 | 18 | 0.76 | 0.36 | 1.63 | 0.4281 |
| AG+AA |  |  | 0.94 | 0.75 | 1.17 |  |
| rs6593206 (IVS1-29869C>A) |  |  |  |  |  |  |
| AA | 309 | 421 | 1.00 |  |  |  |
| AC | 355 | 480 | 1.01 | 0.83 | 1.24 |  |
| CC | 111 | 132 | 1.17 | 0.87 | 1.58 | 0.3796 |
| AC+CC |  |  | 1.05 | 0.86 | 1.27 |  |
| rs759160 (IVS1-28537G>A) |  |  |  |  |  |  |
| AA | 391 | 554 | 1.00 |  |  |  |
| AG | 320 | 411 | 1.12 | 0.92 | 1.36 |  |
| GG | 66 | 69 | 1.40 | 0.97 | 2.02 | 0.0637 |
| AG+GG |  |  | 1.15 | 0.96 | 1.39 |  |
| rs9649847 (IVS1-22841G>A) |  |  |  |  |  |  |
| GG | 551 | 771 | 1.00 |  |  |  |
| AG | 209 | 240 | **1.25** | **1.01** | **1.55** |  |
| AA | 17 | 23 | 1.05 | 0.55 | 2.00 | 0.0855 |
| AG+AA |  |  | 1.23 | 0.99 | 1.52 |  |
| rs6965365 (IVS1-22144T>A) |  |  |  |  |  |  |
| TT | 656 | 855 | 1.00 |  |  |  |
| AT | 116 | 170 | 0.89 | 0.69 | 1.15 |  |
| AA | 3 | 9 | 0.44 | 0.12 | 1.63 | 0.1946 |
| AT+AA |  |  | 0.87 | 0.68 | 1.13 |  |
| rs10488141 (IVS1-14635A>T) |  |  |  |  |  |  |
| AA | 481 | 629 | 1.00 |  |  |  |
| AT | 273 | 360 | 0.96 | 0.79 | 1.17 |  |
| TT | 23 | 46 | 0.65 | 0.38 | 1.09 | 0.2231 |
| AT+TT |  |  | 0.93 | 0.76 | 1.12 |  |
| rs4947979 (IVS1-14354G>A) |  |  |  |  |  |  |
| AA | 491 | 624 | 1.00 |  |  |  |
| AG | 256 | 354 | 0.91 | 0.75 | 1.12 |  |
| GG | 30 | 57 | 0.67 | 0.42 | 1.06 | 0.0957 |
| AG+GG |  |  | 0.88 | 0.73 | 1.07 |  |
| rs10488142 (IVS1-14032C>T) |  |  |  |  |  |  |
| CC | 610 | 796 | 1.00 |  |  |  |
| CT | 150 | 217 | 0.90 | 0.71 | 1.14 |  |
| TT | 17 | 19 | 1.15 | 0.59 | 2.24 | 0.6291 |
| CT+TT |  |  | 0.91 | 0.73 | 1.14 |  |
| rs17151957 (IVS1-9467G>A) |  |  |  |  |  |  |
| GG | 453 | 602 | 1.00 |  |  |  |
| AG | 279 | 380 | 0.95 | 0.78 | 1.16 |  |
| AA | 44 | 53 | 1.09 | 0.71 | 1.66 | 0.9226 |
| AG+AA |  |  | 0.97 | 0.80 | 1.17 |  |
| rs13247687 (IVS1-8203G>A) |  |  |  |  |  |  |
| GG | 238 | 352 | 1.00 |  |  |  |
| AG | 379 | 490 | 1.18 | 0.95 | 1.46 |  |
| AA | 158 | 193 | 1.26 | 0.97 | 1.66 | 0.0705 |
| AG+AA |  |  | 1.21 | 0.99 | 1.48 |  |
| rs6947594 (IVS1-6682A>C) |  |  |  |  |  |  |
| CC | 581 | 747 | 1.00 |  |  |  |
| AC | 181 | 267 | 0.86 | 0.69 | 1.07 |  |
| AA | 15 | 21 | 0.92 | 0.47 | 1.82 | 0.2193 |
| AC+AA |  |  | 0.87 | 0.70 | 1.07 |  |
| rs12666347 (IVS1-4049A>T) |  |  |  |  |  |  |
| AA | 372 | 485 | 1.00 |  |  |  |
| AT | 327 | 452 | 0.97 | 0.80 | 1.18 |  |
| TT | 77 | 98 | 1.05 | 0.76 | 1.46 | 0.9584 |
| AT+TT |  |  | 0.99 | 0.82 | 1.19 |  |
| rs4947984 (IVS3+135G>A) |  |  |  |  |  |  |
| GG | 682 | 899 | 1.00 |  |  |  |
| AG | 93 | 132 | 0.91 | 0.69 | 1.21 |  |
| AA | 0 | 3 | - | - | - | 0.3327 |
| AG+AA |  |  | 0.90 | 0.68 | 1.20 |  |
| rs7801956 (IVS4+10G>A) |  |  |  |  |  |  |
| GG | 670 | 901 | 1.00 |  |  |  |
| AG | 100 | 127 | 1.05 | 0.79 | 1.40 |  |
| AA | 7 | 6 | 1.58 | 0.52 | 4.76 | 0.5115 |
| AG+AA |  |  | 1.07 | 0.81 | 1.40 |  |
| rs2075109 (IVS4-84T>C) |  |  |  |  |  |  |
| CC | 212 | 278 | 1.00 |  |  |  |
| CT | 386 | 550 | 0.95 | 0.76 | 1.19 |  |
| TT | 179 | 207 | 1.18 | 0.90 | 1.54 | 0.2916 |
| CT+TT |  |  | 1.01 | 0.82 | 1.25 |  |
| rs11760524 (IVS6-537A>G) |  |  |  |  |  |  |
| AA | 590 | 776 | 1.00 |  |  |  |
| AG | 175 | 241 | 0.93 | 0.75 | 1.17 |  |
| GG | 12 | 18 | 0.86 | 0.41 | 1.81 | 0.4773 |
| AG+GG |  |  | 0.93 | 0.75 | 1.15 |  |
| rs4947986 (IVS6-49G>A) |  |  |  |  |  |  |
| GG | 447 | 556 | 1.00 |  |  |  |
| AG | 281 | 418 | 0.85 | 0.70 | 1.04 |  |
| AA | 49 | 60 | 1.03 | 0.69 | 1.54 | 0.3379 |
| AG+AA |  |  | 0.87 | 0.72 | 1.05 |  |
| rs1558544 (IVS12+22A>T) |  |  |  |  |  |  |
| TT | 386 | 559 | 1.00 |  |  |  |
| AT | 334 | 403 | 1.20 | 0.99 | 1.47 |  |
| AA | 56 | 71 | 1.17 | 0.80 | 1.70 | 0.0939 |
| AT+AA |  |  | 1.20 | 0.99 | 1.44 |  |
| rs12538371 (IVS15-196T>C) |  |  |  |  |  |  |
| TT | 595 | 802 | 1.00 |  |  |  |
| CT | 175 | 211 | 1.10 | 0.87 | 1.38 |  |
| CC | 7 | 21 | 0.43 | 0.18 | 1.03 | 0.813 |
| CT+CC |  |  | 1.03 | 0.83 | 1.29 |  |
| rs845550 (IVS16+446A>G) |  |  |  |  |  |  |
| GG | 585 | 832 | 1.00 |  |  |  |
| AG | 179 | 187 | **1.39** | **1.10** | **1.76** |  |
| AA | 13 | 16 | 1.17 | 0.56 | 2.48 | **0.0109** |
| AG+AA |  |  | **1.38** | **1.10** | **1.73** |  |
| rs845551 (IVS16-717A>G) |  |  |  |  |  |  |
| GG | 456 | 654 | 1.00 |  |  |  |
| AG | 269 | 323 | **1.24** | **1.01** | **1.52** |  |
| AA | 44 | 48 | 1.33 | 0.87 | 2.05 | **0.0271** |
| AG+AA |  |  | **1.24** | **1.03** | **1.51** |  |
| rs9692301 (IVS21+1241A>G) |  |  |  |  |  |  |
| AA | 357 | 474 | 1.00 |  |  |  |
| AG | 347 | 446 | 1.03 | 0.85 | 1.26 |  |
| GG | 73 | 113 | 0.84 | 0.61 | 1.17 | 0.5545 |
| AG+GG |  |  | 0.99 | 0.82 | 1.19 |  |
| rs9642391 (IVS21+2851G>C) |  |  |  |  |  |  |
| GG | 408 | 525 | 1.00 |  |  |  |
| CG | 310 | 431 | 0.93 | 0.76 | 1.13 |  |
| CC | 59 | 79 | 0.94 | 0.65 | 1.35 | 0.4892 |
| CG+CC |  |  | 0.93 | 0.77 | 1.12 |  |
| rs845552 (IVS21+2994A>G) |  |  |  |  |  |  |
| AA | 219 | 283 | 1.00 |  |  |  |
| AG | 385 | 508 | 0.96 | 0.77 | 1.20 |  |
| GG | 173 | 241 | 0.91 | 0.70 | 1.19 | 0.4904 |
| AG+GG |  |  | 0.94 | 0.76 | 1.16 |  |
| rs845558 (IVS21-1398G>A) |  |  |  |  |  |  |
| GG | 245 | 331 | 1.00 |  |  |  |
| AG | 393 | 487 | 1.10 | 0.89 | 1.37 |  |
| AA | 139 | 216 | 0.85 | 0.65 | 1.12 | 0.3972 |
| AG+AA |  |  | 1.02 | 0.84 | 1.25 |  |
| rs13222385 (IVS22+2422A>G) |  |  |  |  |  |  |
| AA | 304 | 412 | 1.00 |  |  |  |
| AG | 364 | 473 | 1.04 | 0.85 | 1.28 |  |
| GG | 109 | 148 | 1.01 | 0.75 | 1.35 | 0.8437 |
| AG+GG |  |  | 1.03 | 0.85 | 1.25 |  |
| rs845561 (IVS22+3537C>T) |  |  |  |  |  |  |
| TT | 451 | 619 | 1.00 |  |  |  |
| CT | 283 | 347 | 1.14 | 0.93 | 1.40 |  |
| CC | 42 | 66 | 0.90 | 0.60 | 1.36 | 0.6032 |
| CT+CC |  |  | 1.10 | 0.91 | 1.33 |  |
| rs6593210 (IVS22+5015G>A) |  |  |  |  |  |  |
| GG | 468 | 616 | 1.00 |  |  |  |
| AG | 267 | 371 | 0.93 | 0.76 | 1.14 |  |
| AA | 41 | 45 | 1.18 | 0.76 | 1.84 | 0.9648 |
| AG+AA |  |  | 0.95 | 0.79 | 1.16 |  |
| rs845562 (IVS22-4607A>G) |  |  |  |  |  |  |
| GG | 555 | 761 | 1.00 |  |  |  |
| AG | 203 | 244 | 1.17 | 0.94 | 1.46 |  |
| AA | 18 | 26 | 0.96 | 0.52 | 1.77 | 0.29 |
| AG+AA |  |  | 1.14 | 0.92 | 1.40 |  |
| rs6970262 (IVS23+196A>G) |  |  |  |  |  |  |
| GG | 292 | 374 | 1.00 |  |  |  |
| AG | 357 | 490 | 0.92 | 0.75 | 1.13 |  |
| AA | 126 | 171 | 0.91 | 0.69 | 1.21 | 0.4319 |
| AG+AA |  |  | 0.92 | 0.76 | 1.12 |  |
| rs17518446 (IVS24+2433G>A) |  |  |  |  |  |  |
| GG | 548 | 771 | 1.00 |  |  |  |
| AG | 215 | 247 | **1.24** | **1.00** | **1.54** |  |
| AA | 13 | 15 | 1.24 | 0.58 | 2.64 | **0.0528** |
| AG+AA |  |  | **1.24** | **1.00** | **1.52** |  |
| rs2472520 (IVS24-470G>C) |  |  |  |  |  |  |
| CC | 288 | 343 | 1.00 |  |  |  |
| CG | 368 | 509 | 0.86 | 0.70 | 1.06 |  |
| GG | 108 | 171 | 0.76 | 0.57 | 1.01 | **0.0447** |
| CG+GG |  |  | 0.84 | 0.69 | 1.02 |  |
| rs1140475 (Ex25+8T>C) |  |  |  |  |  |  |
| CC | 609 | 801 | 1.00 |  |  |  |
| CT | 157 | 221 | 0.94 | 0.74 | 1.18 |  |
| TT | 10 | 13 | 0.96 | 0.42 | 2.22 | 0.5995 |
| CT+TT |  |  | 0.94 | 0.75 | 1.18 |  |
| rs2293348 (IVS25+201C>T) |  |  |  |  |  |  |
| CC | 355 | 479 | 1.00 |  |  |  |
| CT | 347 | 456 | 1.02 | 0.84 | 1.25 |  |
| TT | 75 | 99 | 1.02 | 0.73 | 1.42 | 0.8526 |
| CT+TT |  |  | 1.02 | 0.85 | 1.23 |  |
| rs2280653 (Ex30+1064A>G) |  |  |  |  |  |  |
| AA | 574 | 748 | 1.00 |  |  |  |
| AG | 185 | 262 | 0.92 | 0.74 | 1.14 |  |
| GG | 18 | 24 | 1.07 | 0.57 | 2.01 | 0.6145 |
| AG+GG |  |  | 0.93 | 0.75 | 1.15 |  |
| rs884419 (Ex30+1250G>A) |  |  |  |  |  |  |
| GG | 665 | 865 | 1.00 |  |  |  |
| AG | 103 | 165 | 0.82 | 0.63 | 1.08 |  |
| AA | 9 | 5 | 2.30 | 0.76 | 6.94 | 0.516 |
| AG+AA |  |  | 0.87 | 0.67 | 1.12 |  |
| rs940806 (6434bp 3' of STP A>G) |  |  |  |  |  |  |
| GG | 221 | 302 | 1.00 |  |  |  |
| AG | 390 | 530 | 1.01 | 0.82 | 1.26 |  |
| AA | 165 | 200 | 1.11 | 0.85 | 1.45 | 0.4843 |
| AG+AA |  |  | 1.04 | 0.84 | 1.28 |  |
|  |  |  |  |  |  |  |
| ***IGFBP3*** |  |  |  |  |  |  |
| rs2471553 (-17383C>T ) |  |  |  |  |  |  |
| AA | 317 | 426 | 1.00 |  |  |  |
| AG | 347 | 469 | 1.00 | 0.81 | 1.22 |  |
| GG | 112 | 136 | 1.12 | 0.84 | 1.50 | 0.5434 |
| AG+GG |  |  | 1.02 | 0.84 | 1.24 |  |
| rs10235181 (-13318A>T ) |  |  |  |  |  |  |
| TT | 752 | 1009 | 1.00 |  |  |  |
| AT | 20 | 21 | 1.17 | 0.63 | 2.18 |  |
| rs13232606 (-10370T>C ) |  |  |  |  |  |  |
| AA | 713 | 953 | 1.00 |  |  |  |
| AG | 61 | 72 | 1.11 | 0.78 | 1.59 |  |
| rs2453836 (-5055T>C ) |  |  |  |  |  |  |
| AA | 485 | 691 | 1.00 |  |  |  |
| AG | 243 | 301 | 1.15 | 0.93 | 1.41 |  |
| GG | 42 | 36 | **1.71** | **1.08** | **2.72** | **0.0206** |
| AG+GG |  |  | **1.21** | **1.00** | **1.47** |  |
| rs903889 (-4255C>A ) |  |  |  |  |  |  |
| TT | 493 | 636 | 1.00 |  |  |  |
| GT | 253 | 342 | 0.97 | 0.79 | 1.18 |  |
| GG | 31 | 57 | 0.73 | 0.46 | 1.15 | 0.2832 |
| GT+GG |  |  | 0.93 | 0.77 | 1.13 |  |
| rs924140 (-2374G>A) |  |  |  |  |  |  |
| CC | 254 | 334 | 1.00 |  |  |  |
| CT | 383 | 531 | 0.92 | 0.75 | 1.14 |  |
| TT | 139 | 165 | 1.07 | 0.81 | 1.42 | 0.8158 |
| CT+TT |  |  | 0.95 | 0.78 | 1.17 |  |
| rs2471551 (IVS1-17C>G) |  |  |  |  |  |  |
| CC | 511 | 694 | 1.00 |  |  |  |
| CG | 262 | 304 | 1.16 | 0.95 | 1.43 |  |
| GG | 46 | 40 | **1.65** | **1.06** | **2.58** | **0.017** |
| CG+GG |  |  | 1.16 | 0.97 | 1.38 |  |
| rs9282734 (Ex2+70A>C ) |  |  |  |  |  |  |
| AA | 789 | 1011 | 1.00 |  |  |  |
| AC | 7 | 6 | 1.45 | 0.48 | 4.35 |  |
| rs3110697 ( IVS3-485T>C) |  |  |  |  |  |  |
| GG | 260 | 362 | 1.00 |  |  |  |
| AG | 382 | 509 | 1.05 | 0.85 | 1.29 |  |
| AA | 134 | 161 | 1.21 | 0.91 | 1.60 | 0.2198 |
| AG+AA |  |  | 1.08 | 0.89 | 1.32 |  |
| rs6670 (Ex5-411A>T) |  |  |  |  |  |  |
| TT | 514 | 740 | 1.00 |  |  |  |
| AT | 227 | 261 | **1.24** | **1.00** | **1.53** |  |
| AA | 30 | 31 | 1.40 | 0.84 | 2.36 | **0.026** |
| AT+AA |  |  | **1.27** | **1.04** | **1.56** |  |
| rs6670 (Ex5-411A>T) |  |  |  |  |  |  |
| GG | 506 | 702 | 1.00 |  |  |  |
| AG | 247 | 294 | 1.15 | 0.94 | 1.42 |  |
| AA | 24 | 37 | 0.87 | 0.51 | 1.49 | 0.4556 |
| AG+AA |  |  | 1.11 | 0.91 | 1.36 |  |
| rs2270628 (4848bp 3' of STPG>A) |  |  |  |  |  |  |
| CC | 514 | 676 | 1.00 |  |  |  |
| CT | 230 | 319 | 0.94 | 0.77 | 1.16 |  |
| TT | 27 | 34 | 1.04 | 0.62 | 1.76 | 0.7289 |
| CT+TT |  |  | 0.95 | 0.78 | 1.16 |  |
| rs12671484 (7263bp 3' of STPT>C) |  |  |  |  |  |  |
| AA | 558 | 726 | 1.00 |  |  |  |
| AG | 186 | 265 | 0.92 | 0.74 | 1.14 |  |
| GG | 13 | 26 | 0.64 | 0.32 | 1.26 | 0.1975 |
| AG+GG |  |  | 0.92 | 0.75 | 1.13 |  |
| rs12702181 (8949bp 3' of STPT>C) |  |  |  |  |  |  |
| AA | 284 | 352 | 1.00 |  |  |  |
| AG | 358 | 509 | 0.89 | 0.72 | 1.09 |  |
| GG | 135 | 173 | 0.96 | 0.73 | 1.27 | 0.5823 |
| AG+GG |  |  | 0.90 | 0.74 | 1.10 |  |
|  |  |  |  |  |  |  |
| ***IGFBP5*** |  |  |  |  |  |  |
| rs17824343 (-20375C>G ) |  |  |  |  |  |  |
| GG | 515 | 659 | 1.00 |  |  |  |
| CG | 230 | 334 | 0.88 | 0.72 | 1.08 |  |
| CC | 30 | 42 | 0.92 | 0.57 | 1.50 | 0.2759 |
| CG+CC |  |  | 0.89 | 0.73 | 1.09 |  |
| rs2024486 (-19806G>C ) |  |  |  |  |  |  |
| CC | 674 | 920 | 1.00 |  |  |  |
| CG | 94 | 112 | 1.15 | 0.86 | 1.54 |  |
| GG | 5 | 2 | 3.37 | 0.65 | 17.51 | 0.1646 |
| CG+GG |  |  | 1.23 | 0.92 | 1.63 |  |
| rs7602780 (-19424G>A ) |  |  |  |  |  |  |
| CC | 610 | 841 | 1.00 |  |  |  |
| CT | 156 | 182 | 1.17 | 0.92 | 1.49 |  |
| TT | 6 | 9 | 0.96 | 0.34 | 2.73 | 0.2628 |
| CT+TT |  |  | 1.17 | 0.93 | 1.48 |  |
| rs2072544 (-17691A>G ) |  |  |  |  |  |  |
| TT | 277 | 362 | 1.00 |  |  |  |
| CT | 358 | 511 | 0.93 | 0.76 | 1.15 |  |
| CC | 142 | 160 | 1.15 | 0.88 | 1.52 | 0.4974 |
| CT+CC |  |  | 0.98 | 0.81 | 1.20 |  |
| rs888184 (-11267C>T ) |  |  |  |  |  |  |
| AA | 543 | 763 | 1.00 |  |  |  |
| AG | 209 | 255 | 1.17 | 0.94 | 1.45 |  |
| GG | 25 | 17 | **2.00** | **1.06** | **3.75** | **0.0235** |
| AG+GG |  |  | 1.22 | 0.99 | 1.50 |  |
| rs10932674 (-9155G>C ) |  |  |  |  |  |  |
| GG | 443 | 561 | 1.00 |  |  |  |
| CG | 286 | 408 | 0.89 | 0.73 | 1.09 |  |
| CC | 44 | 63 | 0.88 | 0.58 | 1.32 | 0.2503 |
| CG+CC |  |  | 0.90 | 0.74 | 1.08 |  |
| rs888186 (-8219C>T ) |  |  |  |  |  |  |
| AA | 653 | 858 | 1.00 |  |  |  |
| AG | 118 | 170 | 0.93 | 0.72 | 1.20 |  |
| GG | 6 | 7 | 1.08 | 0.36 | 3.25 | 0.6438 |
| AG+GG |  |  | 0.93 | 0.73 | 1.20 |  |
| rs6727330 (-3946G>A ) |  |  |  |  |  |  |
| TT | 318 | 462 | 1.00 |  |  |  |
| CT | 365 | 466 | 1.14 | 0.93 | 1.39 |  |
| CC | 93 | 107 | 1.24 | 0.91 | 1.70 | 0.1098 |
| CT+CC |  |  | 1.16 | 0.96 | 1.40 |  |
| rs3770472 (IVS1+427A>G ) |  |  |  |  |  |  |
| TT | 438 | 578 | 1.00 |  |  |  |
| CT | 256 | 367 | 0.91 | 0.74 | 1.12 |  |
| CC | 44 | 56 | 1.05 | 0.69 | 1.59 | 0.6518 |
| CT+CC |  |  | 0.97 | 0.80 | 1.17 |  |
| rs11575134 ( IVS1+3338G>A) |  |  |  |  |  |  |
| CC | 507 | 685 | 1.00 |  |  |  |
| CT | 244 | 313 | 1.05 | 0.86 | 1.29 |  |
| TT | 26 | 37 | 0.95 | 0.56 | 1.59 | 0.8164 |
| CT+TT |  |  | 1.04 | 0.85 | 1.27 |  |
| rs3755137 ( IVS1+5388A>T) |  |  |  |  |  |  |
| TT | 495 | 664 | 1.00 |  |  |  |
| AT | 250 | 330 | 1.05 | 0.86 | 1.29 |  |
| AA | 32 | 41 | 1.07 | 0.66 | 1.74 | 0.5909 |
| AT+AA |  |  | 1.06 | 0.87 | 1.28 |  |
| rs7420849 (IVS1-6727A>C) |  |  |  |  |  |  |
| GG | 272 | 319 | 1.00 |  |  |  |
| GT | 355 | 548 | **0.76** | **0.62** | **0.94** |  |
| TT | 150 | 167 | 1.01 | 0.77 | 1.33 | 0.5737 |
| GT+TT |  |  | **0.82** | **0.67** | **1.00** |  |
| rs7565131 (IVS1-2123T>G) |  |  |  |  |  |  |
| AA | 682 | 926 | 1.00 |  |  |  |
| AC | 92 | 107 | 1.13 | 0.84 | 1.53 |  |
| CC | 1 | 0 | - | - | - | 0.3346 |
| AC+CC |  |  | 1.15 | 0.86 | 1.54 |  |
| rs2241199 ( Ex4-1989A>G) |  |  |  |  |  |  |
| TT | 723 | 956 | 1.00 |  |  |  |
| CT | 54 | 79 | 0.88 | 0.61 | 1.26 |  |
| rs3276 (Ex4-171C>T) |  |  |  |  |  |  |
| GG | 707 | 959 | 1.00 |  |  |  |
| AG | 69 | 76 | 1.21 | 0.86 | 1.70 |  |
| rs4674107 (5119bp 3' of STP T>G) |  |  |  |  |  |  |
| AA | 372 | 507 | 1.00 |  |  |  |
| AC | 342 | 433 | 1.11 | 0.91 | 1.35 |  |
| CC | 63 | 94 | 0.93 | 0.66 | 1.32 | 0.7758 |
| AC+CC |  |  | 1.08 | 0.89 | 1.30 |  |
| rs2067039 ( 7581bp 3' of STP A>T) |  |  |  |  |  |  |
| TT | 770 | 981 | 1.00 |  |  |  |
| AT | 47 | 61 | 0.97 | 0.65 | 1.44 |  |
| rs9282736 ( 7592bp 3' of STP A>T) |  |  |  |  |  |  |
| AA | 817 | 1044 | 1.00 | - | - |  |
|  |  |  |  |  |  |  |
| ***PPARG*** |  |  |  |  |  |  |
| rs2960420 (-78579C>G ) |  |  |  |  |  |  |
| CC | 332 | 419 | 1.00 |  |  |  |
| CG | 335 | 465 | 0.91 | 0.74 | 1.12 |  |
| GG | 108 | 150 | 0.95 | 0.71 | 1.27 | 0.545 |
| CG+GG |  |  | 0.92 | 0.76 | 1.12 |  |
| rs4279078 (-78245G>A ) |  |  |  |  |  |  |
| GG | 619 | 832 | 1.00 |  |  |  |
| AG | 148 | 190 | 1.05 | 0.83 | 1.34 |  |
| AA | 7 | 12 | 0.79 | 0.31 | 2.04 | 0.8873 |
| AG+AA |  |  | 1.05 | 0.83 | 1.33 |  |
| rs2920499 (-74033A>G ) |  |  |  |  |  |  |
| AA | 231 | 279 | 1.00 |  |  |  |
| AG | 365 | 496 | 0.90 | 0.72 | 1.13 |  |
| GG | 180 | 259 | 0.86 | 0.66 | 1.11 | 0.2417 |
| AG+GG |  |  | 0.89 | 0.72 | 1.09 |  |
| rs2920500 (-69678G>A ) |  |  |  |  |  |  |
| AA | 236 | 333 | 1.00 |  |  |  |
| AG | 361 | 492 | 1.03 | 0.83 | 1.28 |  |
| GG | 180 | 210 | 1.18 | 0.91 | 1.53 | 0.2357 |
| AG+GG |  |  | 1.08 | 0.88 | 1.32 |  |
| rs17793951 (IVS3+16785A>G ) |  |  |  |  |  |  |
| AA | 383 | 521 | 1.00 |  |  |  |
| AG | 323 | 428 | 1.02 | 0.83 | 1.24 |  |
| GG | 71 | 85 | 1.11 | 0.78 | 1.56 | 0.6273 |
| AG+GG |  |  | 1.03 | 0.85 | 1.24 |  |
| rs1801282 (Ex4-49C>G ) |  |  |  |  |  |  |
| CC | 588 | 697 | 1.00 |  |  |  |
| CG | 210 | 312 | **0.81** | **0.65** | **0.99** |  |
| GG | 16 | 33 | 0.62 | 0.33 | 1.14 | **0.0149** |
| CG+GG |  |  | **0.84** | **0.71** | **1.00** |  |
| rs1899951 (IVS4+1667C>T) |  |  |  |  |  |  |
| CC | 546 | 683 | 1.00 |  |  |  |
| CT | 207 | 313 | 0.84 | 0.68 | 1.03 |  |
| TT | 17 | 30 | 0.74 | 0.40 | 1.37 | 0.0659 |
| CT+TT |  |  | 0.83 | 0.68 | 1.02 |  |
| rs4135247 (IVS4+3415G>A ) |  |  |  |  |  |  |
| AA | 272 | 380 | 1.00 |  |  |  |
| AG | 387 | 503 | 1.08 | 0.88 | 1.32 |  |
| GG | 117 | 151 | 1.08 | 0.81 | 1.44 | 0.5117 |
| AG+GG |  |  | 1.08 | 0.89 | 1.31 |  |
| rs12629751 (IVS4+6234C>T ) |  |  |  |  |  |  |
| CC | 613 | 829 | 1.00 |  |  |  |
| CT | 157 | 193 | 1.10 | 0.87 | 1.39 |  |
| TT | 6 | 11 | 0.77 | 0.28 | 2.11 | 0.6184 |
| CT+TT |  |  | 1.08 | 0.85 | 1.36 |  |
| rs2120825 (IVS4-7864T>G ) |  |  |  |  |  |  |
| TT | 568 | 717 | 1.00 |  |  |  |
| GT | 195 | 291 | 0.85 | 0.69 | 1.06 |  |
| GG | 13 | 27 | 0.64 | 0.33 | 1.26 | 0.0633 |
| GT+GG |  |  | 0.84 | 0.68 | 1.03 |  |
| rs4135263 (IVS6+276T>C ) |  |  |  |  |  |  |
| TT | 561 | 767 | 1.00 |  |  |  |
| CT | 194 | 242 | 1.12 | 0.90 | 1.39 |  |
| CC | 13 | 21 | 0.85 | 0.42 | 1.73 | 0.5483 |
| CT+CC |  |  | 1.12 | 0.91 | 1.38 |  |
| rs2938392 (IVS7+357G>A ) |  |  |  |  |  |  |
| AA | 203 | 271 | 1.00 |  |  |  |
| AG | 407 | 530 | 1.02 | 0.81 | 1.28 |  |
| GG | 204 | 234 | 1.15 | 0.88 | 1.50 | 0.3 |
| AG+GG |  |  | 1.05 | 0.86 | 1.30 |  |
| rs4135268 (IVS7+2986C>G ) |  |  |  |  |  |  |
| CC | 674 | 904 | 1.00 |  |  |  |
| CG | 97 | 126 | 1.04 | 0.78 | 1.39 |  |
| GG | 4 | 4 | 1.38 | 0.34 | 5.63 | 0.6665 |
| CG+GG |  |  | 1.07 | 0.81 | 1.41 |  |
| rs4135275 ( IVS7-3537A>G) |  |  |  |  |  |  |
| AA | 521 | 675 | 1.00 |  |  |  |
| AG | 225 | 322 | 0.91 | 0.74 | 1.12 |  |
| GG | 28 | 36 | 1.00 | 0.60 | 1.67 | 0.4849 |
| AG+GG |  |  | 0.92 | 0.76 | 1.12 |  |
| rs796313 (IVS8+1948G>T ) |  |  |  |  |  |  |
| GG | 197 | 274 | 1.00 |  |  |  |
| GT | 393 | 530 | 1.01 | 0.81 | 1.27 |  |
| TT | 187 | 231 | 1.11 | 0.85 | 1.45 | 0.4533 |
| GT+TT |  |  | 1.04 | 0.84 | 1.29 |  |
| rs709157 (IVS9+3371G>A ) |  |  |  |  |  |  |
| GG | 409 | 569 | 1.00 |  |  |  |
| AG | 309 | 403 | 1.05 | 0.86 | 1.27 |  |
| AA | 56 | 56 | 1.39 | 0.94 | 2.06 | 0.186 |
| AG+AA |  |  | 1.08 | 0.89 | 1.30 |  |
| rs7645903 (IVS9+5173A>T ) |  |  |  |  |  |  |
| AA | 534 | 721 | 1.00 |  |  |  |
| AT | 224 | 288 | 1.05 | 0.85 | 1.30 |  |
| TT | 17 | 25 | 0.91 | 0.48 | 1.70 | 0.8093 |
| AT+TT |  |  | 1.05 | 0.85 | 1.28 |  |
| rs1797912 ( IVS9-5158A>C) |  |  |  |  |  |  |
| AA | 321 | 451 | 1.00 |  |  |  |
| AC | 361 | 462 | 1.09 | 0.89 | 1.33 |  |
| CC | 95 | 121 | 1.09 | 0.80 | 1.49 | 0.4252 |
| AC+CC |  |  | 1.09 | 0.90 | 1.31 |  |
| rs7626560 (IVS9-309C>T  ) |  |  |  |  |  |  |
| CC | 535 | 738 | 1.00 |  |  |  |
| CT | 227 | 269 | 1.15 | 0.93 | 1.42 |  |
| TT | 14 | 28 | 0.67 | 0.35 | 1.28 | 0.6605 |
| CT+TT |  |  | 1.11 | 0.90 | 1.36 |  |
| rs3856806 (Ex10+161C>T ) |  |  |  |  |  |  |
| CC | 565 | 747 | 1.00 |  |  |  |
| CT | 198 | 262 | 1.01 | 0.82 | 1.26 |  |
| TT | 12 | 25 | 0.70 | 0.35 | 1.41 | 0.6966 |
| CT+TT |  |  | 0.99 | 0.81 | 1.23 |  |
| rs1152003 (Ex10+1213G>C ) |  |  |  |  |  |  |
| CC | 334 | 454 | 1.00 |  |  |  |
| CG | 356 | 462 | 1.03 | 0.85 | 1.26 |  |
| GG | 87 | 119 | 0.97 | 0.71 | 1.32 | 0.9817 |
| CG+GG |  |  | 1.02 | 0.84 | 1.23 |  |
| rs9833097 (Ex10+2975G>A) |  |  |  |  |  |  |
| GG | 597 | 790 | 1.00 |  |  |  |
| AG | 168 | 227 | 0.97 | 0.77 | 1.22 |  |
| AA | 12 | 17 | 0.92 | 0.43 | 1.96 | 0.7342 |
| AG+AA |  |  | 0.96 | 0.77 | 1.20 |  |
| rs4498025 (-49924C>T) |  |  |  |  |  |  |
| CC | 422 | 609 | 1.00 |  |  |  |
| CT | 309 | 356 | 1.26 | 1.04 | 1.54 |  |
| TT | 45 | 69 | 0.97 | 0.65 | 1.45 | 0.16 |
| CT+TT |  |  | 1.22 | 1.01 | 1.47 |  |
| rs1152004 (g.-48195A>G) |  |  |  |  |  |  |
| AA | 542 | 722 | 1.00 |  |  |  |
| AG | 210 | 274 | 1.01 | 0.82 | 1.25 |  |
| GG | 25 | 35 | 0.88 | 0.52 | 1.49 | 0.84 |
| AG+GG |  |  | 0.98 | 0.80 | 1.20 |  |
| rs13090265 (g.-46314A>G) |  |  |  |  |  |  |
| AA | 572 | 757 | 1.00 |  |  |  |
| AG | 187 | 251 | 0.97 | 0.77 | 1.20 |  |
| GG | 16 | 23 | 0.90 | 0.47 | 1.72 | 0.67 |
| AG+GG |  |  | 0.96 | 0.77 | 1.18 |  |
| rs1797877 (g.-45862T>C) |  |  |  |  |  |  |
| CC | 626 | 836 | 1.00 |  |  |  |
| CT | 138 | 186 | 1.00 | 0.78 | 1.28 |  |
| TT | 12 | 12 | 1.30 | 0.58 | 2.93 | 0.76 |
| CT+TT |  |  | 1.02 | 0.80 | 1.29 |  |
|  |  |  |  |  |  |  |
| ***TGFA*** |  |  |  |  |  |  |
| TGFA_42, -15439C>T ( rs713111 ) |  |  |  |  |  |  |
| GG | 577 | 760 | 1.00 |  |  |  |
| AG | 177 | 247 | 0.95 | 0.76 | 1.19 |  |
| AA | 19 | 26 | 0.95 | 0.52 | 1.74 | 0.6741 |
| AG+AA |  |  | 0.97 | 0.78 | 1.20 |  |
| TGFA_40, -15357T>C (rs2215021 ) |  |  |  |  |  |  |
| AA | 312 | 424 | 1.00 |  |  |  |
| AG | 353 | 483 | 0.99 | 0.81 | 1.22 |  |
| GG | 112 | 128 | 1.18 | 0.88 | 1.59 | 0.4011 |
| AG+GG |  |  | 1.03 | 0.85 | 1.25 |  |
| TGFA_36, -14171G>A (rs12996390 ) |  |  |  |  |  |  |
| CC | 627 | 867 | 1.00 |  |  |  |
| CT | 143 | 157 | 1.26 | 0.98 | 1.62 |  |
| TT | 5 | 9 | 0.81 | 0.27 | 2.47 | 0.1334 |
| CT+TT |  |  | 1.24 | 0.97 | 1.58 |  |
| TGFA_39, -8632T>G (rs17040266 ) |  |  |  |  |  |  |
| AA | 473 | 616 | 1.00 |  |  |  |
| AC | 259 | 363 | 0.93 | 0.76 | 1.14 |  |
| CC | 45 | 55 | 1.04 | 0.69 | 1.57 | 0.708 |
| AC+CC |  |  | 0.94 | 0.78 | 1.14 |  |
| TGFA_03, -3762G>C (rs13392762 ) |  |  |  |  |  |  |
| CC | 488 | 641 | 1.00 |  |  |  |
| CG | 250 | 347 | 0.94 | 0.77 | 1.16 |  |
| GG | 39 | 45 | 1.09 | 0.70 | 1.71 | 0.8763 |
| CG+GG |  |  | 0.96 | 0.79 | 1.16 |  |
| TGFA_31, -2927A>G (rs7605323 ) |  |  |  |  |  |  |
| TT | 418 | 546 | 1.00 |  |  |  |
| CT | 293 | 413 | 0.92 | 0.76 | 1.12 |  |
| CC | 66 | 75 | 1.14 | 0.80 | 1.63 | 0.9852 |
| CT+CC |  |  | 0.95 | 0.79 | 1.15 |  |
| TGFA_01, IVS1+779G>C (rs11466191 ) |  |  |  |  |  |  |
| CC | 426 | 557 | 1.00 |  |  |  |
| CG | 298 | 411 | 0.94 | 0.77 | 1.14 |  |
| GG | 53 | 60 | 1.11 | 0.75 | 1.64 | 0.9268 |
| CG+GG |  |  | 0.94 | 0.78 | 1.14 |  |
| TGFA_06, IVS1+4579A>G (rs17639251 ) |  |  |  |  |  |  |
| TT | 586 | 782 | 1.00 |  |  |  |
| CT | 170 | 232 | 1.00 | 0.79 | 1.25 |  |
| CC | 21 | 21 | 1.33 | 0.72 | 2.47 | 0.6374 |
| CT+CC |  |  | 1.03 | 0.82 | 1.28 |  |
| TGFA_34, IVS1+5775T>C (rs4852650 ) |  |  |  |  |  |  |
| AA | 223 | 289 | 1.00 |  |  |  |
| AG | 377 | 530 | 0.92 | 0.74 | 1.15 |  |
| GG | 176 | 216 | 1.04 | 0.80 | 1.36 | 0.8435 |
| AG+GG |  |  | 0.96 | 0.78 | 1.18 |  |
| TGFA_04, IVS1+11495T>G (rs1523300 ) |  |  |  |  |  |  |
| AA | 264 | 359 | 1.00 |  |  |  |
| AC | 360 | 490 | 1.00 | 0.81 | 1.24 |  |
| CC | 153 | 186 | 1.14 | 0.87 | 1.49 | 0.4009 |
| AC+CC |  |  | 1.04 | 0.85 | 1.27 |  |
| TGFA_11, IVS1+13835G>A (rs377122 ) |  |  |  |  |  |  |
| TT | 266 | 324 | 1.00 |  |  |  |
| CT | 370 | 528 | 0.86 | 0.70 | 1.07 |  |
| CC | 140 | 183 | 0.95 | 0.72 | 1.25 | 0.4996 |
| CT+CC |  |  | 0.89 | 0.73 | 1.08 |  |
| TGFA_16, IVS1+17955C>T (rs378322 ) |  |  |  |  |  |  |
| GG | 539 | 726 | 1.00 |  |  |  |
| AG | 213 | 279 | 1.04 | 0.84 | 1.29 |  |
| AA | 24 | 29 | 1.10 | 0.63 | 1.93 | 0.6225 |
| AG+AA |  |  | 1.05 | 0.86 | 1.29 |  |
| TGFA_21, IVS1-17117A>G (rs428225 ) |  |  |  |  |  |  |
| TT | 305 | 407 | 1.00 |  |  |  |
| CT | 361 | 497 | 0.96 | 0.79 | 1.18 |  |
| CC | 111 | 130 | 1.12 | 0.84 | 1.51 | 0.6413 |
| CT+CC |  |  | 0.99 | 0.82 | 1.21 |  |
| TGFA_30, IVS1-16079T>C (rs7562947 ) |  |  |  |  |  |  |
| AA | 621 | 842 | 1.00 |  |  |  |
| AG | 146 | 181 | 1.11 | 0.87 | 1.42 |  |
| GG | 9 | 10 | 1.20 | 0.48 | 2.99 | 0.364 |
| AG+GG |  |  | 1.11 | 0.88 | 1.41 |  |
| TGFA_12, IVS1-14247G>C (rs3771485 ) |  |  |  |  |  |  |
| CC | 494 | 652 | 1.00 |  |  |  |
| CG | 237 | 333 | 0.94 | 0.77 | 1.16 |  |
| GG | 44 | 49 | 1.18 | 0.77 | 1.81 | 0.9253 |
| CG+GG |  |  | 0.98 | 0.80 | 1.19 |  |
| TGFA_22, IVS1-14013G>T (rs446086 ) |  |  |  |  |  |  |
| CC | 451 | 608 | 1.00 |  |  |  |
| AC | 275 | 362 | 1.05 | 0.86 | 1.28 |  |
| AA | 50 | 64 | 1.04 | 0.71 | 1.54 | 0.6815 |
| AC+AA |  |  | 1.05 | 0.86 | 1.26 |  |
| TGFA_29, IVS1-10830C>G (rs6738666 ) |  |  |  |  |  |  |
| GG | 667 | 884 | 1.00 |  |  |  |
| CG | 106 | 145 | 0.99 | 0.75 | 1.30 |  |
| CC | 4 | 6 | 0.87 | 0.24 | 3.12 | 0.8729 |
| CG+CC |  |  | 0.98 | 0.75 | 1.29 |  |
| TGFA_24, IVS1-4682A>G (rs455125 ) |  |  |  |  |  |  |
| TT | 488 | 655 | 1.00 |  |  |  |
| CT | 253 | 337 | 1.00 | 0.82 | 1.23 |  |
| CC | 36 | 43 | 1.15 | 0.72 | 1.82 | 0.7173 |
| CT+CC |  |  | 1.02 | 0.84 | 1.24 |  |
| TGFA_23, IVS1-2063A>G (rs450419 ) |  |  |  |  |  |  |
| TT | 336 | 469 | 1.00 |  |  |  |
| CT | 345 | 459 | 1.03 | 0.85 | 1.26 |  |
| CC | 96 | 107 | 1.24 | 0.91 | 1.70 | 0.2459 |
| CT+CC |  |  | 1.07 | 0.89 | 1.30 |  |
| TGFA_05, IVS2+3441C>G (rs17638303 ) |  |  |  |  |  |  |
| GG | 373 | 532 | 1.00 |  |  |  |
| CG | 333 | 409 | 1.14 | 0.94 | 1.39 |  |
| CC | 71 | 93 | 1.10 | 0.78 | 1.54 | 0.2647 |
| CG+CC |  |  | 1.13 | 0.94 | 1.37 |  |
| TGFA_10, IVS2+4103A>C (rs375668 ) |  |  |  |  |  |  |
| TT | 470 | 630 | 1.00 |  |  |  |
| GT | 261 | 350 | 1.00 | 0.81 | 1.22 |  |
| GG | 44 | 52 | 1.15 | 0.75 | 1.76 | 0.7064 |
| GT+GG |  |  | 1.01 | 0.84 | 1.23 |  |
| TGFA_20, IVS2+8465A>G (rs404420 ) |  |  |  |  |  |  |
| CC | 388 | 461 | 1.00 |  |  |  |
| CT | 319 | 471 | **0.82** | **0.67** | **0.99** |  |
| TT | 67 | 103 | 0.78 | 0.56 | 1.10 | **0.0367** |
| CT+TT |  |  | **0.82** | **0.68** | **0.99** |  |
| TGFA_07, IVS2+9139T>C (rs3755377 ) |  |  |  |  |  |  |
| AA | 242 | 344 | 1.00 |  |  |  |
| AG | 385 | 493 | 1.11 | 0.89 | 1.37 |  |
| GG | 149 | 198 | 1.08 | 0.82 | 1.42 | 0.4921 |
| AG+GG |  |  | 1.10 | 0.90 | 1.35 |  |
| TGFA_17, IVS2+12738T>G (rs3821261 ) |  |  |  |  |  |  |
| AA | 690 | 917 | 1.00 |  |  |  |
| AC | 81 | 113 | 0.96 | 0.71 | 1.31 |  |
| CC | 5 | 4 | 1.52 | 0.41 | 5.72 | 0.97 |
| AC+CC |  |  | 0.99 | 0.73 | 1.33 |  |
| TGFA_13, IVS2+16639T>C (rs3771494 ) |  |  |  |  |  |  |
| AA | 486 | 677 | 1.00 |  |  |  |
| AG | 252 | 324 | 1.10 | 0.89 | 1.34 |  |
| GG | 37 | 33 | 1.55 | 0.95 | 2.53 | 0.0938 |
| AG+GG |  |  | 1.14 | 0.94 | 1.39 |  |
| TGFA_38, IVS2+18827T>C (rs11466225 ) |  |  |  |  |  |  |
| AA | 345 | 424 | 1.00 |  |  |  |
| AG | 325 | 480 | 0.83 | 0.68 | 1.02 |  |
| GG | 107 | 129 | 1.04 | 0.77 | 1.40 | 0.5972 |
| AG+GG |  |  | 0.87 | 0.72 | 1.06 |  |
| TGFA_08, IVS2+22527G>C (rs3755379 ) |  |  |  |  |  |  |
| CC | 712 | 935 | 1.00 |  |  |  |
| CG | 63 | 97 | 0.87 | 0.62 | 1.21 |  |
| GG | 1 | 1 | 1.14 | 0.07 | 18.33 | 0.4279 |
| CG+GG |  |  | 0.86 | 0.62 | 1.20 |  |
| TGFA_09, IVS2+22971G>C (rs3755380 ) |  |  |  |  |  |  |
| GG | 245 | 320 | 1.00 |  |  |  |
| CG | 370 | 512 | 0.93 | 0.75 | 1.16 |  |
| CC | 161 | 202 | 1.03 | 0.78 | 1.34 | 0.9521 |
| CG+CC |  |  | 0.96 | 0.78 | 1.18 |  |
| TGFA_37, IVS2-19137A>G (rs3771504 ) |  |  |  |  |  |  |
| CC | 320 | 409 | 1.00 |  |  |  |
| CT | 344 | 487 | 0.91 | 0.74 | 1.11 |  |
| TT | 112 | 138 | 1.03 | 0.77 | 1.38 | 0.8285 |
| CT+TT |  |  | 0.93 | 0.77 | 1.13 |  |
| TGFA_33, IVS2-11162G>C (rs958686 ) |  |  |  |  |  |  |
| CC | 222 | 274 | 1.00 |  |  |  |
| CG | 352 | 505 | 0.85 | 0.68 | 1.07 |  |
| GG | 200 | 247 | 1.00 | 0.77 | 1.30 | 0.9551 |
| CG+GG |  |  | 0.90 | 0.73 | 1.11 |  |
| TGFA_14, IVS2-4799C>T (rs3771514 ) |  |  |  |  |  |  |
| GG | 451 | 621 | 1.00 |  |  |  |
| AG | 280 | 365 | 1.04 | 0.86 | 1.28 |  |
| AA | 44 | 49 | 1.20 | 0.78 | 1.85 | 0.4161 |
| AG+AA |  |  | 1.07 | 0.89 | 1.30 |  |
| TGFA_15, IVS2-2694G>A (rs3771515 ) |  |  |  |  |  |  |
| CC | 586 | 794 | 1.00 |  |  |  |
| CT | 175 | 221 | 1.06 | 0.85 | 1.33 |  |
| TT | 15 | 20 | 0.99 | 0.50 | 1.95 | 0.6897 |
| CT+TT |  |  | 1.06 | 0.85 | 1.32 |  |
| TGFA_27, IVS3+449T>C (rs6709121 ) |  |  |  |  |  |  |
| GG | 722 | 939 | 1.00 |  |  |  |
| AG | 53 | 90 | 0.81 | 0.57 | 1.16 |  |
| AA | 2 | 4 | 0.57 | 0.10 | 3.14 | 0.1884 |
| AG+AA |  |  | 0.78 | 0.55 | 1.10 |  |
| TGFA_28, IVS3+2614C>G (rs6729950 ) |  |  |  |  |  |  |
| GG | 568 | 745 | 1.00 |  |  |  |
| CG | 191 | 267 | 0.95 | 0.76 | 1.17 |  |
| CC | 18 | 21 | 1.10 | 0.58 | 2.09 | 0.7819 |
| CG+CC |  |  | 0.95 | 0.77 | 1.17 |  |
| TGFA_32, IVS3-831T>C (rs930655 ) |  |  |  |  |  |  |
| GG | 271 | 363 | 1.00 |  |  |  |
| AG | 358 | 491 | 0.98 | 0.79 | 1.21 |  |
| AA | 148 | 179 | 1.10 | 0.84 | 1.45 | 0.5753 |
| AG+AA |  |  | 1.01 | 0.83 | 1.23 |  |
| TGFA_18, IVS4-1190T>C (rs3821272 ) |  |  |  |  |  |  |
| AA | 395 | 497 | 1.00 |  |  |  |
| AG | 303 | 433 | 0.88 | 0.72 | 1.07 |  |
| GG | 79 | 105 | 0.96 | 0.69 | 1.32 | 0.3841 |
| AG+GG |  |  | 0.89 | 0.74 | 1.08 |  |
| TGFA_02, Ex6-1292A>C (rs11466297 ) |  |  |  |  |  |  |
| TT | 621 | 783 | 1.00 |  |  |  |
| GT | 144 | 237 | **0.77** | **0.61** | **0.98** |  |
| GG | 12 | 15 | 0.99 | 0.46 | 2.13 | 0.0648 |
| GT+GG |  |  | **0.79** | **0.63** | **0.99** |  |
| TGFA_35, 9508bp 3' of STP C>G (rs12328204 ) |  |  |  |  |  |  |
| GG | 290 | 354 | 1.00 |  |  |  |
| CG | 356 | 511 | 0.86 | 0.70 | 1.06 |  |
| CC | 131 | 170 | 0.94 | 0.71 | 1.24 | 0.4168 |
| CG+CC |  |  | 0.88 | 0.72 | 1.07 |  |
| TGFA_41, 10865bp 3' of STP A>G (rs549386 ) |  |  |  |  |  |  |
| TT | 295 | 421 | 1.00 |  |  |  |
| CT | 352 | 462 | 1.08 | 0.88 | 1.33 |  |
| CC | 130 | 151 | 1.21 | 0.92 | 1.61 | 0.1778 |
| CT+CC |  |  | 1.11 | 0.92 | 1.35 |  |
| ***VCAM1*** |  |  |  |  |  |  |
| VCAM1_52, -17295C>T (rs1591474) |  |  |  |  |  |  |
| CC | 334 | 447 | 1.00 |  |  |  |
| CT | 351 | 476 | 0.98 | 0.80 | 1.20 |  |
| TT | 92 | 111 | 1.11 | 0.81 | 1.52 | 0.6875 |
| CT+TT |  |  | 1.01 | 0.83 | 1.21 |  |
| VCAM1_92, -16473A>G (rs12568082) |  |  |  |  |  |  |
| GG | 697 | 940 | 1.00 |  |  |  |
| AG | 74 | 91 | 1.09 | 0.78 | 1.50 |  |
| AA | 4 | 3 | 1.72 | 0.38 | 7.76 | 0.4671 |
| AG+AA |  |  | 1.12 | 0.82 | 1.54 |  |
| VCAM1_45, -16088A>G (rs10875328 ) |  |  |  |  |  |  |
| GG | 288 | 375 | 1.00 |  |  |  |
| AG | 368 | 525 | 0.92 | 0.75 | 1.12 |  |
| AA | 119 | 135 | 1.16 | 0.87 | 1.56 | 0.613 |
| AG+AA |  |  | 0.97 | 0.80 | 1.18 |  |
| VCAM1_55, -9765T>C (rs2050471) |  |  |  |  |  |  |
| TT | 218 | 309 | 1.00 |  |  |  |
| CT | 396 | 528 | 1.07 | 0.86 | 1.33 |  |
| CC | 163 | 198 | 1.16 | 0.89 | 1.53 | 0.2769 |
| CT+CC |  |  | 1.10 | 0.89 | 1.35 |  |
| VCAM1_90, -3840A>G (rs4584412 ) |  |  |  |  |  |  |
| AA | 528 | 732 | 1.00 |  |  |  |
| AG | 221 | 272 | 1.12 | 0.91 | 1.39 |  |
| GG | 27 | 30 | 1.27 | 0.74 | 2.17 | 0.1873 |
| AG+GG |  |  | 1.14 | 0.93 | 1.40 |  |
| VCAM1_57, IVS2+912A>G (rs3176860) |  |  |  |  |  |  |
| AA | 235 | 325 | 1.00 |  |  |  |
| AG | 403 | 516 | 1.09 | 0.88 | 1.35 |  |
| GG | 139 | 193 | 0.99 | 0.75 | 1.31 | 0.9064 |
| AG+GG |  |  | 1.06 | 0.86 | 1.30 |  |
| VCAM1_08, IVS3+514C>T (rs3917009) |  |  |  |  |  |  |
| CC | 641 | 810 | 1.00 |  |  |  |
| CT | 124 | 204 | **0.76** | **0.60** | **0.98** |  |
| TT | 11 | 18 | 0.81 | 0.38 | 1.73 | **0.0401** |
| CT+TT |  |  | **0.76** | **0.60** | **0.97** |  |
| VCAM1_73, IVS4+420A>C (rs3917010 ) |  |  |  |  |  |  |
| AA | 442 | 559 | 1.00 |  |  |  |
| AC | 282 | 413 | 0.86 | 0.71 | 1.05 |  |
| CC | 53 | 62 | 1.08 | 0.73 | 1.59 | 0.4889 |
| AC+CC |  |  | 0.89 | 0.74 | 1.07 |  |
| VCAM1_61, IVS4-458C>T (rs3176867 ) |  |  |  |  |  |  |
| CC | 403 | 568 | 1.00 |  |  |  |
| CT | 319 | 391 | 1.16 | 0.96 | 1.42 |  |
| TT | 54 | 74 | 1.02 | 0.70 | 1.48 | 0.3245 |
| CT+TT |  |  | 1.14 | 0.94 | 1.37 |  |
| VCAM1_68, IVS7+468C>T (rs3181088) |  |  |  |  |  |  |
| CC | 541 | 739 | 1.00 |  |  |  |
| CT | 210 | 276 | 1.06 | 0.86 | 1.31 |  |
| TT | 26 | 20 | **1.84** | **1.01** | **3.36** | 0.1351 |
| CT+TT |  |  | 1.11 | 0.91 | 1.37 |  |
| VCAM1_76, IVS8+823T>A (rs3917016 ) |  |  |  |  |  |  |
| TT | 582 | 782 | 1.00 |  |  |  |
| AT | 174 | 233 | 1.01 | 0.81 | 1.27 |  |
| AA | 19 | 17 | 1.57 | 0.80 | 3.06 | 0.4614 |
| AT+AA |  |  | 1.04 | 0.84 | 1.30 |  |
| VCAM1_67, IVS8-284A>T (rs3176877 ) |  |  |  |  |  |  |
| TT | 288 | 376 | 1.00 |  |  |  |
| AT | 369 | 490 | 0.99 | 0.81 | 1.22 |  |
| AA | 120 | 166 | 0.94 | 0.71 | 1.25 | 0.7273 |
| AT+AA |  |  | 0.98 | 0.80 | 1.19 |  |
| VCAM1_03, Ex9+20C>T (rs3176878 ) |  |  |  |  |  |  |
| CC | 508 | 658 | 1.00 |  |  |  |
| CT | 232 | 323 | 0.93 | 0.76 | 1.15 |  |
| TT | 35 | 51 | 0.89 | 0.57 | 1.39 | 0.4355 |
| CT+TT |  |  | 0.93 | 0.76 | 1.13 |  |
| VCAM1_44, 9495bp 3' of STP A>T (rs10493936 ) |  |  |  |  |  |  |
| AA | 393 | 485 | 1.00 |  |  |  |
| AT | 320 | 443 | 0.89 | 0.73 | 1.09 |  |
| TT | 64 | 106 | 0.75 | 0.54 | 1.06 | 0.0788 |
| AT+TT |  |  | 0.87 | 0.72 | 1.04 |  |
| ***VEGF*** |  |  |  |  |  |  |
| VEGF_41, -19052G>T (rs9394963 ) |  |  |  |  |  |  |
| GG | 346 | 456 | 1.00 |  |  |  |
| GT | 340 | 468 | 0.96 | 0.78 | 1.17 |  |
| TT | 91 | 111 | 1.06 | 0.78 | 1.45 | 0.9415 |
| GT+TT |  |  | 0.98 | 0.81 | 1.18 |  |
| VEGF_35, -15108A>C (rs833052) |  |  |  |  |  |  |
| CC | 575 | 778 | 1.00 |  |  |  |
| AC | 191 | 240 | 1.10 | 0.88 | 1.37 |  |
| AA | 11 | 17 | 0.88 | 0.41 | 1.91 | 0.5766 |
| AC+AA |  |  | 1.08 | 0.87 | 1.34 |  |
| VEGF_39, -11487C>T (rs866236 ) |  |  |  |  |  |  |
| CC | 441 | 611 | 1.00 |  |  |  |
| CT | 285 | 370 | 1.10 | 0.90 | 1.35 |  |
| TT | 51 | 53 | 1.31 | 0.87 | 1.96 | 0.1448 |
| CT+TT |  |  | 1.13 | 0.93 | 1.36 |  |
| VEGF_36, -11413G>T (rs833057 ) |  |  |  |  |  |  |
| TT | 452 | 627 | 1.00 |  |  |  |
| GT | 270 | 345 | 1.12 | 0.92 | 1.37 |  |
| GG | 52 | 52 | 1.38 | 0.92 | 2.06 | 0.0848 |
| GT+GG |  |  | 1.14 | 0.94 | 1.38 |  |
| VEGF_28, -7799A>T ( rs1547651 ) |  |  |  |  |  |  |
| AA | 512 | 710 | 1.00 |  |  |  |
| AT | 235 | 295 | 1.14 | 0.92 | 1.40 |  |
| TT | 28 | 30 | 1.31 | 0.77 | 2.24 | 0.1364 |
| AT+TT |  |  | 1.16 | 0.95 | 1.42 |  |
| VEGF_59, -6589C>T (rs833058 ) |  |  |  |  |  |  |
| CC | 301 | 366 | 1.00 |  |  |  |
| CT | 364 | 483 | 0.91 | 0.74 | 1.11 |  |
| TT | 112 | 183 | **0.73** | **0.55** | **0.97** | **0.0331** |
| CT+TT |  |  | 0.85 | 0.70 | 1.04 |  |
| VEGF_34, -5774A>G (rs699946 ) |  |  |  |  |  |  |
| AA | 530 | 675 | 1.00 |  |  |  |
| AG | 224 | 327 | 0.85 | 0.69 | 1.05 |  |
| GG | 23 | 33 | 0.84 | 0.49 | 1.45 | 0.1254 |
| AG+GG |  |  | 0.85 | 0.70 | 1.04 |  |
| VEGF_37, -3294G>T (rs833060 ) |  |  |  |  |  |  |
| GG | 454 | 579 | 1.00 |  |  |  |
| GT | 271 | 388 | 0.89 | 0.73 | 1.08 |  |
| TT | 51 | 55 | 1.11 | 0.74 | 1.67 | 0.6623 |
| GT+TT |  |  | 0.89 | 0.74 | 1.08 |  |
| VEGF_01, -2054A>C (rs699947) |  |  |  |  |  |  |
| CC | 199 | 278 | 1.00 |  |  |  |
| AC | 400 | 511 | 1.11 | 0.89 | 1.40 |  |
| AA | 212 | 242 | 1.26 | 0.97 | 1.64 | 0.0831 |
| AC+AA |  |  | 1.15 | 0.93 | 1.41 |  |
| VEGF_03, Ex1+398C>G (rs2010963 ) |  |  |  |  |  |  |
| GG | 447 | 543 | 1.00 |  |  |  |
| CG | 341 | 463 | 0.90 | 0.74 | 1.09 |  |
| CC | 77 | 99 | 0.91 | 0.65 | 1.26 | 0.3081 |
| CG+CC |  |  | 0.94 | 0.79 | 1.11 |  |
| VEGF_05, Ex1-73C>T (rs25648 ) |  |  |  |  |  |  |
| CC | 557 | 750 | 1.00 |  |  |  |
| CT | 249 | 296 | 1.16 | 0.95 | 1.42 |  |
| TT | 26 | 25 | 1.46 | 0.83 | 2.57 | 0.0691 |
| CT+TT |  |  | 1.19 | 1.00 | 1.42 |  |
| VEGF_45, IVS2+1378C>T (rs3024994 ) |  |  |  |  |  |  |
| CC | 656 | 871 |  |  |  |  |
| CT | 115 | 154 | 1.00 | 0.77 | 1.31 |  |
| TT | 6 | 4 | 2.04 | 0.57 | 7.29 | 0.6763 |
| CT+TT |  |  | 0.99 | 0.76 | 1.28 |  |
| VEGF_29, IVS5-892T>C (rs3025010 ) |  |  |  |  |  |  |
| TT | 320 | 436 | 1.00 |  |  |  |
| CT | 336 | 475 | 0.98 | 0.80 | 1.20 |  |
| CC | 117 | 122 | **1.36** | **1.01** | **1.83** | 0.1279 |
| CT+CC |  |  | 1.06 | 0.88 | 1.28 |  |
| VEGF_30, IVS7-1203A>G (rs3025033) |  |  |  |  |  |  |
| AA | 518 | 683 | 1.00 |  |  |  |
| AG | 230 | 313 | 0.97 | 0.79 | 1.19 |  |
| GG | 29 | 39 | 0.94 | 0.57 | 1.54 | 0.7085 |
| AG+GG |  |  | 0.97 | 0.79 | 1.18 |  |
| VEGF_31, IVS7-919C>T (rs3025035) |  |  |  |  |  |  |
| CC | 673 | 893 | 1.00 |  |  |  |
| CT | 100 | 135 | 0.97 | 0.74 | 1.29 |  |
| TT | 4 | 7 | 0.73 | 0.21 | 2.54 | 0.7057 |
| CT+TT |  |  | 0.96 | 0.73 | 1.26 |  |
| VEGF_04, Ex8+259C>T (rs3025039 ) |  |  |  |  |  |  |
| CC | 604 | 764 | 1.00 |  |  |  |
| CT | 233 | 295 | 1.01 | 0.83 | 1.24 |  |
| TT | 22 | 36 | 0.73 | 0.43 | 1.27 | 0.5945 |
| CT+TT |  |  | 1.01 | 0.85 | 1.21 |  |
| VEGF_42, 5530bp 3' of STP C>A (rs998584 ) |  |  |  |  |  |  |
| CC | 199 | 300 | 1.00 |  |  |  |
| AC | 390 | 507 | 1.15 | 0.92 | 1.44 |  |
| AA | 188 | 227 | 1.24 | 0.95 | 1.62 | 0.107 |
| AC+AA |  |  | 1.18 | 0.95 | 1.46 |  |
| VEGF_65, 5958bp 3' of STP A>C (rs6899540 ) |  |  |  |  |  |  |
| AA | 530 | 686 | 1.00 |  |  |  |
| AC | 223 | 309 | 0.93 | 0.76 | 1.14 |  |
| CC | 24 | 40 | 0.78 | 0.46 | 1.32 | 0.2945 |
| AC+CC |  |  | 0.91 | 0.75 | 1.12 |  |
| VEGF_33, 6119bp 3' of STP C>T (rs6900017) |  |  |  |  |  |  |
| CC | 674 | 879 | 1.00 |  |  |  |
| CT | 98 | 145 | 0.88 | 0.67 | 1.17 |  |
| TT | 4 | 6 | 0.93 | 0.26 | 3.35 | 0.3984 |
| CT+TT |  |  | 0.86 | 0.66 | 1.13 |  |
| VEGF_64, 6507bp 3' of STP G>A (rs6905288 ) |  |  |  |  |  |  |
| AA | 243 | 308 | 1.00 |  |  |  |
| AG | 389 | 515 | 0.95 | 0.76 | 1.17 |  |
| GG | 145 | 212 | 0.87 | 0.66 | 1.14 | 0.3258 |
| AG+GG |  |  | 0.92 | 0.75 | 1.13 |  |
| VEGF_66, 11448bp 3' of STP A>G (rs879825 ) |  |  |  |  |  |  |
| AA | 690 | 909 | 1.00 |  |  |  |
| AG | 79 | 117 | 0.89 | 0.66 | 1.21 |  |
| GG | 5 | 6 | 1.14 | 0.34 | 3.80 | 0.5659 |
| AG+GG |  |  | 0.91 | 0.68 | 1.22 |  |
| **Apoptosis Genes** |  |  |  |  |  |  |
| **CASP 1/5/4/12** |  |  |  |  |  |  |
| LOC440067_02 (rs1785883) |  |  |  |  |  |  |
| GG | 686 | 880 | 1.00 |  |  |  |
| AG | 85 | 145 | **0.75** | **0.56** | **1.00** |  |
| AA | 2 | 8 | 0.34 | 0.07 | 1.60 | **0.02** |
| AG+AA |  |  | **0.75** | **0.57** | **1.00** |  |
| LOC440067_03 (rs508760) |  |  |  |  |  |  |
| GG | 658 | 893 | 1.00 |  |  |  |
| GT | 112 | 135 | 1.12 | 0.85 | 1.47 |  |
| TT | 4 | 6 | 1.05 | 0.29 | 3.81 | 0.45 |
| GT+TT |  |  | 1.13 | 0.87 | 1.48 |  |
| rs7934239 (-12676T>C ) |  |  |  |  |  |  |
| GG | 700 | 940 | 1.00 |  |  |  |
| AG | 74 | 90 | 1.11 | 0.80 | 1.54 |  |
| AA | 2 | 2 | 1.27 | 0.18 | 9.10 | 0.5025 |
| AG+AA |  |  | 1.09 | 0.79 | 1.50 |  |
| rs501626 (-12291A>G) |  |  |  |  |  |  |
| TT | 580 | 820 | 1.00 |  |  |  |
| CT | 183 | 199 | **1.30** | **1.04** | **1.64** |  |
| CC | 14 | 16 | 1.19 | 0.57 | 2.48 | **0.0343** |
| CT+CC |  |  | **1.29** | **1.04** | **1.62** |  |
| rs11821722 (-11804G>A ) |  |  |  |  |  |  |
| TT | 396 | 548 | 1.00 |  |  |  |
| CT | 310 | 405 | 1.09 | 0.89 | 1.33 |  |
| CC | 70 | 81 | 1.21 | 0.85 | 1.71 | 0.2297 |
| CT+CC |  |  | 1.11 | 0.92 | 1.34 |  |
| rs568910 (IVS2+365T>G) |  |  |  |  |  |  |
| AA | 518 | 744 | 1.00 |  |  |  |
| AC | 225 | 254 | **1.28** | **1.03** | **1.59** |  |
| CC | 31 | 35 | 1.24 | 0.75 | 2.05 | **0.03** |
| AC+CC |  |  | **1.28** | **1.05** | **1.57** |  |
| rs492859 (-5645T>G ) |  |  |  |  |  |  |
| CC | 514 | 741 | 1.00 |  |  |  |
| AC | 227 | 251 | **1.31** | **1.06** | **1.62** |  |
| AA | 30 | 35 | 1.21 | 0.73 | 2.00 | **0.0238** |
| AC+AA |  |  | **1.29** | **1.05** | **1.58** |  |
| rs3181318 (-373 C>T) |  |  |  |  |  |  |
| GG | 337 | 472 | 1.00 |  |  |  |
| AG | 349 | 457 | 1.09 | 0.90 | 1.33 |  |
| AA | 89 | 106 | 1.20 | 0.88 | 1.65 | 0.2063 |
| AG+AA |  |  | 1.12 | 0.93 | 1.35 |  |
| rs507879 (Ex2-118A>G ) |  |  |  |  |  |  |
| TT | 212 | 337 | 1.00 |  |  |  |
| CT | 381 | 481 | 1.29 | **1.03** | **1.60** |  |
| CC | 179 | 209 | 1.39 | **1.07** | **1.82** | **0.0108** |
| CT+CC |  |  | 1.31 | **1.07** | **1.61** |  |
| rs3181175 (IVS2-1151A>G ) |  |  |  |  |  |  |
| TT | 583 | 774 | 1.00 |  |  |  |
| CT | 175 | 234 | 0.99 | 0.79 | 1.24 |  |
| CC | 15 | 20 | 1.00 | 0.51 | 1.98 | 0.96 |
| CT+CC |  |  | 0.99 | 0.79 | 1.22 |  |
| rs9651713 (IVS2-647C>T ) |  |  |  |  |  |  |
| GG | 862 | 652 | 1.00 |  |  |  |
| AG | 164 | 120 | 0.96 | 0.74 | 1.25 |  |
| AA | 8 | 5 | 0.77 | 0.25 | 2.38 | 0.6624 |
| AG+AA |  |  | 0.95 | 0.74 | 1.22 |  |
| rs17446518 (IVS3+451T>A ) |  |  |  |  |  |  |
| AA | 620 | 833 | 1.00 |  |  |  |
| AT | 149 | 194 | 1.03 | 0.81 | 1.30 |  |
| TT | 8 | 8 | 1.49 | 0.55 | 4.06 | 0.62 |
| AT+TT |  |  | 1.04 | 0.82 | 1.32 |  |
| rs540819 (IVS8+6T>A ) |  |  |  |  |  |  |
| AA | 304 | 393 | 1.00 |  |  |  |
| AT | 337 | 477 | 0.92 | 0.75 | 1.13 |  |
| TT | 133 | 162 | 1.03 | 0.78 | 1.36 | 0.9398 |
| AT+TT |  |  | 0.95 | 0.78 | 1.15 |  |
| rs3181338 (1585bp 3' of STP G>T) |  |  |  |  |  |  |
| CC | 593 | 778 | 1.00 |  |  |  |
| AC | 169 | 242 | 0.91 | 0.73 | 1.14 |  |
| AA | 13 | 15 | 1.07 | 0.50 | 2.28 | 0.5213 |
| AC+AA |  |  | 0.93 | 0.75 | 1.16 |  |
| rs4121642 (9404bp 3' of STP C>T) |  |  |  |  |  |  |
| GG | 538 | 703 | 1.00 |  |  |  |
| AG | 214 | 298 | 0.93 | 0.75 | 1.15 |  |
| AA | 25 | 32 | 0.96 | 0.56 | 1.65 | 0.5501 |
| AG+AA |  |  | 0.93 | 0.76 | 1.14 |  |
| rs1941425 (-11609G>C ) |  |  |  |  |  |  |
| GG | 535 | 711 | 1.00 |  |  |  |
| CG | 212 | 296 | 0.95 | 0.77 | 1.17 |  |
| CC | 29 | 26 | 1.48 | 0.86 | 2.56 | 0.6881 |
| CG+CC |  |  | 0.99 | 0.81 | 1.21 |  |
| rs571407 (IVS5+380G>A ) |  |  |  |  |  |  |
| CC | 284 | 399 | 1.00 |  |  |  |
| CT | 366 | 472 | 1.10 | 0.90 | 1.36 |  |
| TT | 127 | 162 | 1.07 | 0.81 | 1.42 | 0.4841 |
| CT+TT |  |  | 1.09 | 0.90 | 1.33 |  |
| rs11226565 (IVS7-374T>C ) |  |  |  |  |  |  |
| AA | 435 | 605 | 1.00 |  |  |  |
| AG | 291 | 372 | 1.09 | 0.89 | 1.32 |  |
| GG | 49 | 57 | 1.17 | 0.78 | 1.75 | 0.3086 |
| AG+GG |  |  | 1.10 | 0.91 | 1.33 |  |
| rs7125155 (IVS8+893A>G ) |  |  |  |  |  |  |
| TT | 681 | 912 | 1.00 |  |  |  |
| CT | 93 | 119 | 1.03 | 0.77 | 1.38 |  |
| CC | 3 | 4 | 1.00 | 0.22 | 4.52 | 0.8618 |
| CT+CC |  |  | 1.03 | 0.77 | 1.37 |  |
| rs547584 (IVS9-187G>A ) |  |  |  |  |  |  |
| TT | 361 | 511 | 1.00 |  |  |  |
| CT | 324 | 417 | 1.10 | 0.90 | 1.34 |  |
| CC | 79 | 96 | 1.13 | 0.81 | 1.57 | 0.3121 |
| CT+CC |  |  | 1.12 | 0.93 | 1.35 |  |
| rs475931 (30025bp 3' of STP T>C) |  |  |  |  |  |  |
| AA | 684 | 909 | 1.00 |  |  |  |
| AG | 92 | 123 | 0.99 | 0.74 | 1.33 |  |
| GG | 1 | 2 | 0.61 | 0.06 | 6.74 | 0.8767 |
| AG+GG |  |  | 0.98 | 0.73 | 1.30 |  |
| rs11825902 |  |  |  |  |  |  |
| CC | 448 | 627 | 1.00 |  |  |  |
| CT | 281 | 350 | 1.13 | 0.92 | 1.38 |  |
| TT | 48 | 58 | 1.13 | 0.75 | 1.69 | 0.25 |
| CT+TT |  |  | 1.13 | 0.93 | 1.36 |  |
| rs571955 (.NC_*6148bp 3' of STP T>C) |  |  |  |  |  |  |
| GG | 316 | 442 | 1.00 |  |  |  |
| AG | 354 | 456 | 1.09 | 0.89 | 1.34 |  |
| AA | 107 | 137 | 1.11 | 0.83 | 1.49 | 0.3726 |
| AG+AA |  |  | 1.10 | 0.91 | 1.33 |  |
| rs655954 (.NC_*8791bp 3' of STP A>G) |  |  |  |  |  |  |
| CC | 613 | 798 | 1.00 |  |  |  |
| CT | 151 | 221 | 0.90 | 0.71 | 1.14 |  |
| TT | 12 | 15 | 1.06 | 0.49 | 2.29 | 0.5043 |
| CT+TT |  |  | 0.91 | 0.73 | 1.15 |  |
| ***CASP2*** |  |  |  |  |  |  |
| rs7806162 |  |  |  |  |  |  |
| CC | 424 | 550 | 1.00 |  |  |  |
| AC | 290 | 400 | 0.94 | 0.77 | 1.15 |  |
| AA | 61 | 84 | 0.91 | 0.64 | 1.3 | 0.47 |
| AC+AA |  |  | 0.94 | 0.78 | 1.13 |  |
| rs3181165 (IVS1-6G>A ) |  |  |  |  |  |  |
| GG | 706 | 926 | 1.00 |  |  |  |
| AG | 67 | 104 | 0.84 | 0.60 | 1.16 |  |
| AA | 3 | 3 | 1.23 | 0.24 | 6.24 | 0.3562 |
| AG+AA |  |  | 0.84 | 0.61 | 1.16 |  |
| rs3181166 (IVS2+149T>G ) |  |  |  |  |  |  |
| TT | 561 | 743 | 1.00 |  |  |  |
| GT | 191 | 262 | 0.96 | 0.77 | 1.19 |  |
| GG | 23 | 25 | 1.17 | 0.65 | 2.09 | 0.9709 |
| GT+GG |  |  | 0.97 | 0.78 | 1.19 |  |
| rs4647321 (IVS10+93T>C) |  |  |  |  |  |  |
| TT | 701 | 949 | 1.00 |  |  |  |
| CT | 75 | 85 | 1.19 | 0.86 | 1.65 | 0.3022 |
| rs7810486 (4946bp 3' of STP A>G ) |  |  |  |  |  |  |
| GG | 408 | 537 | 1.00 |  |  |  |
| AG | 300 | 427 | 0.92 | 0.76 | 1.12 |  |
| AA | 66 | 65 | 1.37 | 0.95 | 1.99 | 0.5404 |
| AG+AA |  |  | 0.98 | 0.81 | 1.18 |  |
| rs10500136 |  |  |  |  |  |  |
| TT | 617 | 838 | 1.00 |  |  |  |
| CT | 154 | 184 | 1.13 | 0.89 | 1.43 |  |
| CC | 6 | 10 | 0.87 | 0.31 | 2.45 | 0.43 |
| CT+CC |  |  | 1.1 | 0.87 | 1.39 |  |
| ***CASP3*** |  |  |  |  |  |  |
| rs4440267 |  |  |  |  |  |  |
| GG | 700 | 947 | 1.00 |  |  |  |
| AG | 75 | 85 | 1.22 | 0.88 | 1.7 |  |
| AA | 2 | 3 | 0.93 | 0.15 | 5.63 | 0.27 |
| AG+AA |  |  | 1.21 | 0.88 | 1.68 |  |
| rs4862401 |  |  |  |  |  |  |
| CC | 335 | 426 | 1.00 |  |  |  |
| CG | 361 | 480 | 0.96 | 0.78 | 1.17 |  |
| GG | 79 | 125 | 0.78 | 0.57 | 1.08 | 0.18 |
| CG+GG |  |  | 0.92 | 0.76 | 1.11 |  |
| rs870825 |  |  |  |  |  |  |
| AA | 573 | 763 | 1.00 |  |  |  |
| AG | 191 | 251 | 1.01 | 0.81 | 1.25 |  |
| GG | 13 | 21 | 0.8 | 0.39 | 1.61 | 0.79 |
| AG+GG |  |  | 0.99 | 0.8 | 1.22 |  |
| rs2720380 |  |  |  |  |  |  |
| AA | 532 | 695 | 1.00 |  |  |  |
| AT | 210 | 303 | 0.89 | 0.72 | 1.1 |  |
| TT | 21 | 28 | 0.95 | 0.53 | 1.7 | 0.35 |
| AT+TT |  |  | 0.93 | 0.76 | 1.13 |  |
| rs2720378 (IVS2+1506G>) |  |  |  |  |  |  |
| CC | 369 | 475 | 1.00 |  |  |  |
| CG | 327 | 437 | 0.96 | 0.78 | 1.17 |  |
| GG | 81 | 120 | 0.83 | 0.61 | 1.14 | 0.2868 |
| CG+GG |  |  | 0.92 | 0.77 | 1.11 |  |
| rs4647610 (IVS2+1688G>A ) |  |  |  |  |  |  |
| CC | 552 | 720 | 1.00 |  |  |  |
| CT | 203 | 282 | 0.92 | 0.75 | 1.15 |  |
| TT | 16 | 26 | 0.77 | 0.41 | 1.46 | 0.3192 |
| CT+TT |  |  | 0.91 | 0.74 | 1.12 |  |
| rs2705901 (IVS3+68C>G ) |  |  |  |  |  |  |
| CC | 562 | 745 | 1.00 |  |  |  |
| CG | 197 | 265 | 0.99 | 0.80 | 1.23 |  |
| GG | 18 | 25 | 0.98 | 0.53 | 1.82 | 0.9118 |
| CG+GG |  |  | 0.99 | 0.80 | 1.22 |  |
| rs2720376 (IVS4-750G>A) |  |  |  |  |  |  |
| TT | 221 | 282 | 1.00 |  |  |  |
| CT | 379 | 490 | 0.96 | 0.76 | 1.20 |  |
| CC | 176 | 263 | 0.83 | 0.64 | 1.08 | 0.1691 |
| CT+CC |  |  | 0.91 | 0.74 | 1.13 |  |
| rs2705897 (IVS5-4A>C ) |  |  |  |  |  |  |
| GG | 405 | 506 | 1.00 |  |  |  |
| GT | 309 | 442 | 0.86 | 0.71 | 1.05 |  |
| TT | 63 | 86 | 0.91 | 0.64 | 1.29 | 0.2146 |
| GT+TT |  |  | 0.87 | 0.72 | 1.04 |  |
| rs1049253 (Ex8-102T>C) |  |  |  |  |  |  |
| AA | 506 | 663 | 1.00 |  |  |  |
| AG | 241 | 342 | 0.91 | 0.74 | 1.11 |  |
| GG | 29 | 28 | 1.34 | 0.79 | 2.30 | 0.881 |
| AG+GG |  |  | 0.94 | 0.77 | 1.14 |  |
| ***CASP6*** |  |  |  |  |  |  |
| rs2285714 |  |  |  |  |  |  |
| CC | 232 | 319 | 1.00 |  |  |  |
| CT | 405 | 504 | 1.11 | 0.90 | 1.38 |  |
| TT | 139 | 211 | 0.92 | 0.70 | 1.20 | 0.7 |
| CT+TT |  |  | 1.05 | 0.86 | 1.29 |  |
| rs1541373 (-4342G>A ) |  |  |  |  |  |  |
| TT | 320 | 447 | 1.00 |  |  |  |
| CT | 372 | 467 | 1.11 | 0.91 | 1.35 |  |
| CC | 84 | 121 | 0.97 | 0.71 | 1.34 | 0.7327 |
| CT+CC |  |  | 1.08 | 0.90 | 1.31 |  |
| rs768063 (IVS1-958A>G ) |  |  |  |  |  |  |
| CC | 744 | 977 | 1.00 |  |  |  |
| CT | 32 | 57 | 0.72 | 0.46 | 1.12 |  |
| TT | 1 | 1 | 1.69 | 0.10 | 28.41 | 0.197 |
| CT+TT |  |  | 0.73 | 0.47 | 1.14 |  |
| rs5030552 (IVS2-82A>G ) |  |  |  |  |  |  |
| TT | 615 | 835 | 1.00 |  |  |  |
| CT | 152 | 190 | 1.04 | 0.82 | 1.33 |  |
| CC | 9 | 9 | 1.36 | 0.53 | 3.45 | 0.5687 |
| CT+CC |  |  | 1.06 | 0.84 | 1.34 |  |
| rs1800627 (IVS5-1493T>C ) |  |  |  |  |  |  |
| AA | 212 | 266 | 1.00 |  |  |  |
| AG | 381 | 512 | 0.93 | 0.74 | 1.17 |  |
| GG | 184 | 257 | 0.91 | 0.70 | 1.19 | 0.4794 |
| AG+GG |  |  | 0.93 | 0.75 | 1.14 |  |
| rs5030606 |  |  |  |  |  |  |
| AA | 237 | 308 | 1.00 |  |  |  |
| AG | 388 | 526 | 0.94 | 0.76 | 1.17 |  |
| GG | 152 | 201 | 0.97 | 0.74 | 1.27 | 0.75 |
| AG+GG |  |  | 0.95 | 0.77 | 1.16 |  |
| rs3733611 |  |  |  |  |  |  |
| CC | 362 | 484 | 1.00 |  |  |  |
| CT | 349 | 463 | 1.00 | 0.82 | 1.21 |  |
| TT | 66 | 87 | 1.04 | 0.73 | 1.47 | 0.91 |
| CT+TT |  |  | 1.00 | 0.83 | 1.21 |  |
| ***CASP7*** |  |  |  |  |  |  |
| rs7907942 |  |  |  |  |  |  |
| CC | 568 | 731 | 1.00 |  |  |  |
| CT | 197 | 276 | 0.92 | 0.74 | 1.14 |  |
| TT | 11 | 27 | 0.53 | 0.26 | 1.08 | 0.14 |
| CT+TT |  |  | 0.89 | 0.72 | 1.10 |  |
| rs17773680 |  |  |  |  |  |  |
| CC | 663 | 863 | 1.00 |  |  |  |
| CT | 107 | 168 | 0.83 | 0.63 | 1.08 |  |
| TT | 5 | 2 | 3.45 | 0.66 | 18.08 | 0.39 |
| CT+TT |  |  | 0.86 | 0.66 | 1.12 |  |
| rs3127126 |  |  |  |  |  |  |
| GG | 352 | 445 | 1.00 |  |  |  |
| AG | 331 | 458 | 0.89 | 0.73 | 1.09 |  |
| AA | 93 | 131 | 0.90 | 0.67 | 1.22 | 0.32 |
| AG+AA |  |  | 0.90 | 0.74 | 1.08 |  |
| rs12358415 |  |  |  |  |  |  |
| CC | 327 | 455 | 1.00 |  |  |  |
| CT | 356 | 451 | 1.10 | 0.90 | 1.35 |  |
| TT | 84 | 117 | 0.99 | 0.72 | 1.37 | 0.68 |
| CT+TT |  |  | 1.08 | 0.90 | 1.31 |  |
| rs10885493 |  |  |  |  |  |  |
| GG | 593 | 761 | 1.00 |  |  |  |
| AG | 173 | 248 | 0.90 | 0.72 | 1.13 |  |
| AA | 9 | 23 | 0.51 | 0.24 | 1.13 | 0.12 |
| AG+AA |  |  | 0.87 | 0.70 | 1.08 |  |
| rs7906704 |  |  |  |  |  |  |
| GG | 484 | 632 | 1.00 |  |  |  |
| GT | 257 | 349 | 0.96 | 0.78 | 1.17 |  |
| TT | 29 | 44 | 0.84 | 0.52 | 1.36 | 0.46 |
| GT+TT |  |  | 0.94 | 0.78 | 1.14 |  |
| rs11196418 (-13264A>G ) |  |  |  |  |  |  |
| GG | 588 | 770 | 1.00 |  |  |  |
| AG | 177 | 245 | 0.96 | 0.77 | 1.20 |  |
| AA | 11 | 19 | 0.74 | 0.35 | 1.57 | 0.4789 |
| AG+AA |  |  | 0.94 | 0.76 | 1.17 |  |
| rs11196422 (IVS2+3353G>A ) |  |  |  |  |  |  |
| GG | 582 | 772 | 1.00 |  |  |  |
| AG | 178 | 239 | 0.98 | 0.79 | 1.23 |  |
| AA | 15 | 21 | 0.87 | 0.45 | 1.72 | 0.7526 |
| AG+AA |  |  | 0.97 | 0.78 | 1.21 |  |
| rs4132820 (IVS4+2846C>T ) |  |  |  |  |  |  |
| CC | 621 | 814 | 1.00 |  |  |  |
| CT | 145 | 202 | 0.96 | 0.75 | 1.22 |  |
| TT | 3 | 12 | 0.32 | 0.09 | 1.15 | 0.2995 |
| CT+TT |  |  | 0.94 | 0.74 | 1.18 |  |
| rs3124741 (IVS4+11446C>T ) |  |  |  |  |  |  |
| CC | 523 | 712 | 1.00 |  |  |  |
| CT | 221 | 284 | 1.05 | 0.85 | 1.29 |  |
| TT | 32 | 37 | 1.20 | 0.73 | 1.96 | 0.4636 |
| CT+TT |  |  | 1.06 | 0.87 | 1.30 |  |
| rs12416109 (IVS6-461G>A ) |  |  |  |  |  |  |
| GG | 197 | 297 | 1.00 |  |  |  |
| AG | 389 | 491 | 1.21 | 0.97 | 1.52 |  |
| AA | 190 | 245 | 1.19 | 0.92 | 1.55 | 0.1756 |
| AG+AA |  |  | 1.20 | 0.97 | 1.49 |  |
| rs4353229 (Ex10+520T>C ) |  |  |  |  |  |  |
| TT | 411 | 562 | 1.00 |  |  |  |
| CT | 305 | 391 | 1.07 | 0.88 | 1.31 |  |
| CC | 61 | 82 | 1.00 | 0.70 | 1.43 | 0.6865 |
| CT+Cc |  |  | 1.06 | 0.88 | 1.28 |  |
| rs10787498 (Ex10+581T>G ) |  |  |  |  |  |  |
| TT | 360 | 437 | 1.00 |  |  |  |
| GT | 325 | 474 | 0.84 | 0.69 | 1.03 |  |
| GG | 92 | 124 | 0.87 | 0.64 | 1.18 | 0.1576 |
| GT+GG |  |  | 0.85 | 0.70 | 1.03 |  |
| rs1127687 (Ex10-556G>A ) |  |  |  |  |  |  |
| GG | 476 | 654 | 1.00 |  |  |  |
| AG | 268 | 339 | 1.11 | 0.91 | 1.36 |  |
| AA | 33 | 42 | 1.10 | 0.69 | 1.77 | 0.3181 |
| AG+AA |  |  | 1.11 | 0.92 | 1.35 |  |
| rs12358301 (8018bp 3' of STP C>T) |  |  |  |  |  |  |
| TT | 545 | 709 | 1.00 |  |  |  |
| CT | 211 | 292 | 0.96 | 0.77 | 1.18 |  |
| CC | 20 | 32 | 0.79 | 0.44 | 1.40 | 0.4368 |
| CT+CC |  |  | 0.94 | 0.76 | 1.15 |  |
| rs3127077 (11334bp 3' of STP T>C) |  |  |  |  |  |  |
| TT | 532 | 706 | 1.00 |  |  |  |
| CT | 207 | 294 | 0.93 | 0.75 | 1.15 |  |
| CC | 34 | 32 | 1.45 | 0.88 | 2.40 | 0.7303 |
| CT+CC |  |  | 0.98 | 0.81 | 1.20 |  |
| ***CASP8/10*** |  |  |  |  |  |  |
| rs12613347 (IVS3-2395C>T ) |  |  |  |  |  |  |
| CC | 459 | 613 | 1.00 |  |  |  |
| CT | 274 | 367 | 0.98 | 0.80 | 1.19 |  |
| TT | 44 | 54 | 1.08 | 0.71 | 1.64 | 0.9376 |
| CT+TT |  |  | 0.99 | 0.82 | 1.20 |  |
| rs3731714 (IVS5+149C>T) |  |  |  |  |  |  |
| CC | 410 | 542 | 1.00 |  |  |  |
| CT | 297 | 422 | 0.93 | 0.76 | 1.13 |  |
| TT | 67 | 71 | 1.25 | 0.87 | 1.80 | 0.7024 |
| CT+TT |  |  | 0.98 | 0.81 | 1.18 |  |
| rs3769825 (IVS2-11399A>G ) |  |  |  |  |  |  |
| GG | 197 | 265 | 1.00 |  |  |  |
| AG | 391 | 518 | 1.00 | 0.79 | 1.25 |  |
| AA | 188 | 250 | 1.00 | 0.77 | 1.31 | 0.9866 |
| AG+AA |  |  | 1.00 | 0.81 | 1.24 |  |
| rs11899004 (IVS2-8753G>A ) |  |  |  |  |  |  |
| GG | 526 | 670 | 1.00 |  |  |  |
| AG | 223 | 326 | 0.88 | 0.71 | 1.08 |  |
| AA | 25 | 34 | 0.91 | 0.53 | 1.55 | 0.2474 |
| AG+AA |  |  | 0.88 | 0.72 | 1.07 |  |
| rs6736233 (IVS2-3805G>C ) |  |  |  |  |  |  |
| GG | 631 | 846 | 1.00 |  |  |  |
| CG | 139 | 177 | 1.07 | 0.84 | 1.37 |  |
| CC | 6 | 10 | 0.79 | 0.28 | 2.19 | 0.7663 |
| CG+CC |  |  | 1.05 | 0.83 | 1.34 |  |
| rs3769821 (IVS3+325C>T ) |  |  |  |  |  |  |
| TT | 318 | 440 | 1.00 |  |  |  |
| CT | 349 | 484 | 0.99 | 0.81 | 1.22 |  |
| CC | 97 | 99 | 1.38 | 1.01 | 1.90 | 0.1467 |
| CT+CC |  |  | 1.07 | 0.88 | 1.29 |  |
| rs2293554 (IVS5+73T>G ) |  |  |  |  |  |  |
| TT | 651 | 864 | 1.00 |  |  |  |
| GT | 119 | 161 | 0.96 | 0.74 | 1.25 |  |
| GG | 5 | 9 | 0.68 | 0.22 | 2.04 | 0.5931 |
| GT+GG |  |  | 0.96 | 0.74 | 1.23 |  |
| rs10931936 (IVS11+2101T>C ) |  |  |  |  |  |  |
| CC | 390 | 542 | 1.00 |  |  |  |
| CT | 319 | 427 | 1.03 | 0.84 | 1.25 |  |
| TT | 64 | 63 | 1.43 | 0.98 | 2.07 | 0.1668 |
| CT+TT |  |  | 1.08 | 0.90 | 1.31 |  |
| rs1045485 (Ex13+51G>C ) |  |  |  |  |  |  |
| GG | 597 | 777 | 1.00 |  |  |  |
| CG | 170 | 242 | 0.91 | 0.73 | 1.14 |  |
| CC | 10 | 16 | 0.78 | 0.35 | 1.75 | 0.3286 |
| CG+CC |  |  | 0.90 | 0.72 | 1.13 |  |
| rs1035140 (1173bp 3' of STP A>T) |  |  |  |  |  |  |
| AA | 229 | 317 | 1.00 |  |  |  |
| AT | 383 | 503 | 1.04 | 0.84 | 1.29 |  |
| TT | 164 | 215 | 1.04 | 0.79 | 1.36 | 0.7653 |
| AT+TT |  |  | 1.04 | 0.85 | 1.28 |  |
| rs700636 (1934bp 3' of STP A>C) |  |  |  |  |  |  |
| CC | 221 | 315 | 1.00 |  |  |  |
| AC | 393 | 526 | 1.08 | 0.87 | 1.34 |  |
| AA | 162 | 192 | 1.22 | 0.93 | 1.60 | 0.1615 |
| AC+AA |  |  | 1.12 | 0.91 | 1.37 |  |
| rs11679181 |  |  |  |  |  |  |
| CC | 254 | 334 | 1.00 |  |  |  |
| CT | 391 | 508 | 1.00 | 0.81 | 1.24 |  |
| TT | 131 | 186 | 0.92 | 0.70 | 1.22 | 0.63 |
| CT+TT |  |  | 0.97 | 0.80 | 1.19 |  |
| ***CASP9*** |  |  |  |  |  |  |
| rs7516435 |  |  |  |  |  |  |
| AA | 382 | 524 | 1.00 |  |  |  |
| AG | 338 | 420 | 1.11 | 0.92 | 1.36 |  |
| GG | 57 | 90 | 0.92 | 0.64 | 1.32 | 0.75 |
| AG+GG |  |  | 1.08 | 0.89 | 1.30 |  |
| rs933704 (IVS1+1868G>A) |  |  |  |  |  |  |
| TT | 235 | 298 | 1.00 |  |  |  |
| CT | 385 | 517 | 0.93 | 0.74 | 1.15 |  |
| CC | 153 | 214 | 0.89 | 0.68 | 1.16 | 0.3651 |
| CT+CC |  |  | 0.91 | 0.74 | 1.12 |  |
| rs2020902 (IVS3+8T>C) |  |  |  |  |  |  |
| AA | 578 | 776 | 1.00 |  |  |  |
| AG | 183 | 242 | 1.01 | 0.81 | 1.26 |  |
| GG | 16 | 17 | 1.27 | 0.63 | 2.55 | 0.7135 |
| AG+GG |  |  | 1.02 | 0.82 | 1.27 |  |
| rs4661636 (IVS6-1114G>A ) |  |  |  |  |  |  |
| CC | 353 | 477 | 1.00 |  |  |  |
| CT | 337 | 446 | 1.00 | 0.82 | 1.23 |  |
| TT | 80 | 107 | 0.99 | 0.71 | 1.36 | 0.9605 |
| CT+TT |  |  | 1.01 | 0.83 | 1.22 |  |
| rs4646092 (IVS7+95G>A ) |  |  |  |  |  |  |
| CC | 427 | 573 | 1.00 |  |  |  |
| CT | 292 | 405 | 0.96 | 0.79 | 1.17 |  |
| TT | 57 | 56 | 1.36 | 0.92 | 2.01 | 0.4644 |
| CT+TT |  |  | 1.01 | 0.84 | 1.22 |  |
| rs12130370 |  |  |  |  |  |  |
| TT | 219 | 297 | 1.00 |  |  |  |
| CT | 384 | 523 | 1.01 | 0.81 | 1.26 |  |
| CC | 172 | 213 | 1.11 | 0.85 | 1.45 | 0.47 |
| CT+CC |  |  | 1.04 | 0.84 | 1.28 |  |
| rs3766160 |  |  |  |  |  |  |
| GG | 439 | 598 | 1.00 |  |  |  |
| AG | 290 | 372 | 1.09 | 0.89 | 1.33 |  |
| AA | 48 | 64 | 1.06 | 0.71 | 1.58 | 0.47 |
| AG+AA |  |  | 1.08 | 0.89 | 1.31 |  |
| ***CASP14*** |  |  |  |  |  |  |
| rs8110862 |  |  |  |  |  |  |
| AA | 340 | 495 | 1.00 |  |  |  |
| AC | 357 | 445 | 1.16 | 0.95 | 1.41 |  |
| CC | 80 | 93 | 1.21 | 0.87 | 1.68 | 0.12 |
| AC+CC |  |  | 1.16 | 0.96 | 1.40 |  |
| rs5021087 |  |  |  |  |  |  |
| GG | 418 | 576 | 1.00 |  |  |  |
| GT | 308 | 382 | 1.08 | 0.89 | 1.32 |  |
| TT | 51 | 75 | 0.91 | 0.62 | 1.32 | 0.9 |
| GT+TT |  |  | 1.05 | 0.87 | 1.26 |  |
| rs714920 (-11344T>C ) |  |  |  |  |  |  |
| TT | 251 | 315 | 1.00 |  |  |  |
| CT | 388 | 533 | 0.93 | 0.75 | 1.15 |  |
| CC | 135 | 186 | 0.92 | 0.70 | 1.22 | 0.503 |
| CT+CC |  |  | 0.93 | 0.76 | 1.14 |  |
| rs4808901 (-5786C>T ) |  |  |  |  |  |  |
| CC | 257 | 362 | 1.00 |  |  |  |
| CT | 363 | 488 | 1.06 | 0.86 | 1.31 |  |
| TT | 157 | 183 | 1.24 | 0.94 | 1.62 | 0.14 |
| CT+TT |  |  | 1.11 | 0.91 | 1.35 |  |
| rs10518246 (-3081A>G) |  |  |  |  |  |  |
| GG | 529 | 738 | 1.00 |  |  |  |
| AG | 227 | 263 | 1.21 | 0.98 | 1.5 |  |
| AA | 21 | 32 | 0.88 | 0.50 | 1.55 | 0.2467 |
| AG+AA |  |  | 1.17 | 0.95 | 1.43 |  |
| rs3181309 (894bp 3' of STP C>T) |  |  |  |  |  |  |
| TT | 486 | 590 | 1.00 |  |  |  |
| CT | 254 | 392 | **0.78** | **0.64** | **0.95** |  |
| CC | 37 | 53 | 0.85 | 0.55 | 1.32 | **0.0336** |
| CT+CC |  |  | **0.79** | **0.65** | **0.96** |  |
| rs10425745 (2935bp 3' of STP A>T) |  |  |  |  |  |  |
| TT | 392 | 558 | 1.00 |  |  |  |
| AT | 330 | 403 | 1.21 | 0.99 | 1.47 |  |
| AA | 55 | 73 | 1.08 | 0.74 | 1.57 | 0.1573 |
| AT+AA |  |  | 1.19 | 0.98 | 1.43 |  |
| rs16980286 (8052bp 3' of STP C>T) |  |  |  |  |  |  |
| TT | 413 | 549 | 1.00 |  |  |  |
| CT | 300 | 408 | 0.99 | 0.81 | 1.20 |  |
| CC | 64 | 78 | 1.10 | 0.77 | 1.57 | 0.8013 |
| CT+CC |  |  | 1.00 | 0.83 | 1.21 |  |

^1^Adjusted for age, gender and center
