# Supplementary material for: An Analysis of Growth, Differentiation and Apoptosis Genes with Risk of Renal Cancer
Source: PLoS One. 2009 Mar 24;4(3):e4895. doi: 10.1371/journal.pone.0004895 (PMC2656573; doi:10.1371/journal.pone.0004895)
Supplement: Table S2 — EGFR integrated haplotype and renal cancer risk. (0.06 MB DOC) [file pone.0004895.s005.doc]

**Supplementary Table 2. *EGFR* integrated haplotype and renal cancer risk**

| EGFR regions 2 and 3 combined | | | | |  |  |  |  |  |  |  |  |  |  |
| --- | --- | --- | --- | --- | --- | --- | --- | --- | --- | --- | --- | --- | --- | --- |
|  |  |  |  |  |  |  |  |  | cases% | controls % | OR^1^ | 95% | CI | p-value |
| haplo.base | C | G | G | A | A | T | G |  | 0.10109 | 0.13382 | 1 |  |  |  |
| geno3.glm.39 | T | G | G | A | A | T | G |  | 0.07988 | 0.07681 | 1.39 | 0.94 | 2.05 | 0.10028 |
| geno3.glm.14 | C | G | G | A | A | G | G |  | 0.09013 | 0.07323 | **1.6** | **1.09** | **2.37** | **0.01743** |
| geno3.glm.40 | T | G | G | A | G | G | G |  | 0.06132 | 0.06303 | 1.25 | 0.86 | 1.83 | 0.24235 |
| geno3.glm.32 | T | A | G | C | A | T | G |  | 0.05849 | 0.06182 | 1.18 | 0.8 | 1.76 | 0.40823 |
| geno3.glm.31 | T | A | G | C | A | T | A |  | 0.0515 | 0.05907 | 1.21 | 0.82 | 1.77 | 0.33315 |
| geno3.glm.28 | T | A | G | A | A | T | G |  | 0.06314 | 0.05894 | 1.43 | 0.96 | 2.14 | 0.07607 |
| geno3.glm.34 | T | G | A | A | G | G | G |  | 0.06928 | 0.04904 | **1.84** | **1.25** | **2.71** | **0.00209** |
| geno3.glm.15 | C | G | G | A | A | T | A |  | 0.05352 | 0.0486 | 1.28 | 0.85 | 1.94 | 0.23626 |
| geno3.glm.30 | T | A | G | C | A | G | G |  | 0.04168 | 0.04327 | 1.29 | 0.82 | 2.04 | 0.27032 |
| geno3.glm.3 | C | A | G | A | A | T | G |  | 0.03413 | 0.03855 | 1.15 | 0.68 | 1.94 | 0.61014 |
| geno3.glm.18 | C | G | G | A | G | G | G |  | 0.04275 | 0.03658 | 1.43 | 0.91 | 2.27 | 0.12422 |
| geno3.glm.4 | C | A | G | C | A | G | G |  | 0.03458 | 0.03525 | 1.21 | 0.74 | 2 | 0.4523 |
| geno3.glm.36 | T | G | A | A | G | T | G |  | 0.03918 | 0.03117 | 1.6 | 0.97 | 2.64 | 0.06338 |
| geno3.glm.10 | C | G | A | A | G | G | G |  | 0.0336 | 0.03067 | 1.49 | 0.85 | 2.59 | 0.15977 |
| geno3.glm.26 | T | A | G | A | A | G | G |  | 0.02263 | 0.0287 | 1.03 | 0.57 | 1.88 | 0.91371 |
| geno3.glm.1 | C | A | G | A | A | G | G |  | 0.02061 | 0.0265 | 0.97 | 0.52 | 1.83 | 0.92857 |
| geno3.glm.12 | C | G | A | A | G | T | G |  | 0.02582 | 0.02633 | 1.26 | 0.68 | 2.32 | 0.4664 |
| geno3.glm.37 | T | G | G | A | A | G | G |  | 0.01404 | 0.02308 | 0.66 | 0.3 | 1.44 | 0.29511 |
| geno3.glm.rare | * | * | * | * | * | * | * |  |  |  | **1.55** | **1.02** | **2.36** | **0.04145** |
|  |  |  |  |  |  |  |  |  |  |  |  |  |  |  |
| Global p-value |  |  |  |  |  |  |  |  |  |  |  |  |  | 0.08351 |

^1^Adjusted for age, sex, and center
